# SPOP mutation induces DNA methylation via stabilizing GLP/G9a

**DOI:** 10.1038/s41467-021-25951-3

**Published:** 2021-09-29

**Authors:** Jianong Zhang, Kun Gao, Hongyan Xie, Dejie Wang, Pingzhao Zhang, Ting Wei, Yuqian Yan, Yunqian Pan, Wenbin Ye, Huifen Chen, Qing Shi, Yao Li, Shi-min Zhao, Xiaonan Hou, Saravut J. Weroha, Yuzhuo Wang, Jun Zhang, R. Jeffrey Karnes, Housheng Hansen He, Liguo Wang, Chenji Wang, Haojie Huang

**Affiliations:** 1 State Key Lab of Genetic Engineering, MOE Engineering Research Center of Gene Technology, School of Life Sciences, Fudan University, 200438 Shanghai, China; 2 Department of Biochemistry and Molecular Biology, Mayo Clinic College of Medicine and Science, Rochester, MN 55905 USA; 3 Department of Clinical Laboratory, Shanghai First Maternity and Infant Hospital, School of Medicine, Tongji University, 200092 Shanghai, China; 4 Fudan University Shanghai Cancer Center and Department of Pathology, Shanghai Medical College, Fudan University, 200032 Shanghai, China; 5 Divison of Computational Biology, Mayo Clinic College of Medicine and Science, Rochester, MN 55905 USA; 6 Department of Automation, Xiamen University, Xiamen, Fujian China; 7 Department of Medical Biophysics, University of Toronto, Toronto, ON Canada; 8 Princess Margaret Cancer Centre, University Health Network, Toronto, ON Canada; 9 Department of Oncology, Mayo Clinic College of Medicine, Rochester, MN 55905 USA; 10 Department of Experimental Therapeutics, BC Cancer Research Centre, Vancouver, BC Canada; 11 Department of Laboratory Medicine and Pathology, Mayo Clinic College of Medicine and Science, Scottsdale, AZ 85259 USA; 12 Department of Urology, Mayo Clinic College of Medicine and Science, Rochester, MN 55905 USA; 13 Mayo Clinic Cancer Center, Mayo Clinic College of Medicine and Science, Rochester, MN 55905 USA

**Keywords:** Ubiquitylation, Prostate cancer, DNA methylation

## Abstract

Mutations in *SPOP* E3 ligase gene are reportedly associated with genome-wide DNA hypermethylation in prostate cancer (PCa) although the underlying mechanisms remain elusive. Here, we demonstrate that SPOP binds and promotes polyubiquitination and degradation of histone methyltransferase and DNMT interactor GLP. SPOP mutation induces stabilization of GLP and its partner protein G9a and aberrant upregulation of global DNA hypermethylation in cultured PCa cells and primary PCa specimens. Genome-wide DNA methylome analysis shows that a subset of tumor suppressor genes (TSGs) including *FOXO3*, *GATA5*, and *NDRG1*, are hypermethylated and downregulated in SPOP-mutated PCa cells. DNA methylation inhibitor 5-azacytidine effectively reverses expression of the TSGs examined, inhibits SPOP-mutated PCa cell growth in vitro and in mice, and enhances docetaxel anti-cancer efficacy. Our findings reveal the GLP/G9a-DNMT module as a mediator of DNA hypermethylation in SPOP-mutated PCa. They suggest that SPOP mutation could be a biomarker for effective treatment of PCa with DNA methylation inhibitor alone or in combination with taxane chemotherapeutics.

## Abstract (web summary)

The molecular mechanism underlying the DNA hypermethylation phenotype observed in the SPOP-mutant prostate cancers is unclear. Here, the authors show that mutant SPOP induces global aberrant DNA methylation patterns through GLP/G9a and renders prostate cancer cells susceptible to DNA demethylating agents.

## Introduction

Aberrant epigenetic alterations are frequently observed in almost all types of cancer and are known to cooperate with genetic alterations to drive cancer initiation and progression^1–3^. Among various epigenetic changes, DNA hypermethylation dramatically alters genome structure and gene expression profiles during oncogenesis^4^. Increased promoter methylation of tumor suppressor genes (TSGs) has been linked to oncogenesis and therapy resistance in many cancer types because promoter hypermethylation often leads to epigenetic silencing of TSGs^5^. Several groups have performed DNA methylation profiling in PCa at both primary and metastatic stages^3,6–9^. Notably, SPOP-mutated primary tumors associate with global DNA hypermethylation compared to other genetic subtypes^3^. DNA hypermethylation in IDH1-mutated tumors at both primary and metastatic stages^3,7^ can be explained by the increased production of 2-hydroxyglutatate (2HG), an antagonist of TET dioxygenases^10^. However, the underlying mechanisms of DNA hypermethylation in SPOP-mutated PCa remain unknown.

SPOP is a substrate-binding adaptor of the CULLIN3 (CUL3)-RBX1 E3 ubiquitin ligase complex (CRL3^SPOP^)^11,12^. The *SPOP* gene is frequently mutated in PCa, accounting for approximately 10% of primary PCa across demographically diverse patient cohorts^3,13^. The vast majority of SPOP mutations detected in PCa occur in the MATH domain involved in substrate binding^14,15^. As a result, SPOP mutations often cause aberrant accumulation of its substrates which include PCa-relevant proteins such as androgen receptor (AR), BRD4, SRC-3, TRIM24, ERG, PD-L1, and c-MYC^16–21^.

GLP (encoded by *EHMT1*) and G9a (encoded by *EHMT2*) form a protein complex and function as a euchromatic histone methyltransferase (HMTase) to catalyze mono- and di-methylation of histone H3K9 (H3K9me1/2), resulting in epigenetic silencing of target genes^22,23^. The GLP/G9a complex has also been reported to promote gene silencing by inducing DNA hypermethylation in catalytic activity-dependent and -independent manners^24–26^. H3K9 methylation contributes to the formation of heterochromatin and DNA methylation by recruiting other epigenetic factors such as heterochromatin protein 1 (HP1)^27^. In a manner independent of histone methylation, GLP/G9a mediate methylation of LIG1, which subsequently recruit UHRF1 and DNMT1 to maintain DNA methylation during DNA replication^28^. The GLP/G9a complex can also induce DNA methylation by functioning as a scaffold to recruit DNMT proteins^24^. The GLP/G9a complex is reportedly involved in a number of biological processes including tumor cell growth and metastasis^29,30^. G9a and GLP are frequently overexpressed in several cancers, and enzymatic inhibitors of GLP/G9a have been developed and proved effective in the inhibition of the growth of certain cancer types in vitro and in vivo^31,32^, representing a promising therapeutic agent in cancer treatment. However, the function and regulation of GLP/G9a complex in PCa remain poorly understood.

To identify the molecular mediator(s) of DNA hypermethylation in SPOP-mutant PCa cells, we performed yeast two-hybrid (Y2H) screen and identified GLP as a novel binding partner of SPOP. We demonstrate that SPOP binds to and promotes ubiquitination and proteasomal degradation of GLP. In contrast, mutations in SPOP result in elevation of GLP and its partner protein G9a, DNA hypermethylation and silencing of a subset of TSGs. Treatment of the DNA methylation inhibitor 5-azacytidine not only reverses these processes, but also sensitizes SPOP-mutated PCa cells to the first-line chemotherapy agent taxane.

## Results

### SPOP-mutant expression increases global DNA methylation in PCa cells

**Fig. 1:**
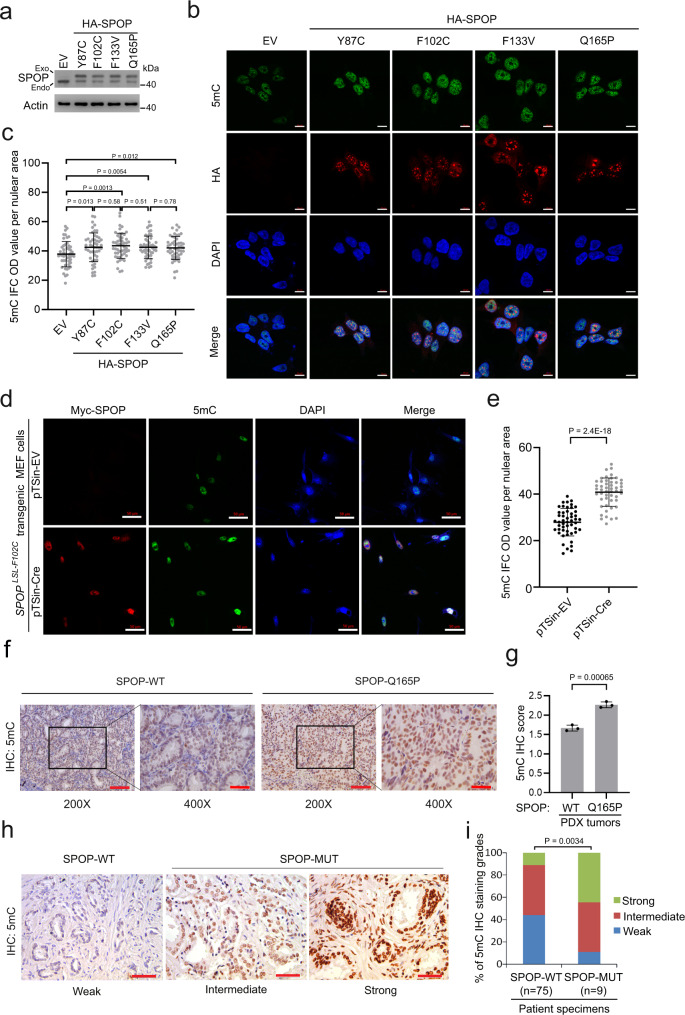
SPOP-mutant expression induces DNA hypermethylation in cultured PCa cells and patient specimens. **a**–**c** Western blots of whole cell lysate (WCL) from 22Rv1 cells infected with lentivirus expressing empty vector (EV), HA-tagged SPOP Y87C, F102C, F133V or Q165P mutant (**a**). Representative IFC images of 5mC and HA-SPOP staining are shown in (**b**) and 5mC signals were quantified using ImageJ optical density (OD)/nuclear area (pixel) (**c**). Data shown means ± SD (*n* = 50 cells/group). Scale bar, 10 μm. **d**, **e** Representative IFC images of Myc-SPOP F102C and 5mC staining in MEFs (derived from Myc-SPOP^LSL^ conditional transgenic mice) infected with the indicated lentivirus (**d**) and 5mC signals were quantified using ImageJ optical density (OD)/nuclear area (pixel) (**e**). Data shown means ± SD (*n* = 50 cells/group). Scale bar, 50 μm. **f**, **g** Representative IHC images of 5mC staining in SPOP WT and SPOP Q165P mutant PDX tissues (**f**) and the quantitative data of 5mC staining (**g**). Scale bar in 200 × field, 100 μm. Scale bar in 400 × field, 50 μm. Data shown means ± SD (*n* = 3 replicates/group). **h**, **i** Representative IHC images of 5mC staining in 84 PCa patient specimens including 75 SPOP-WT and 9 SPOP-mutant (MUT) samples (**h**) and the quantitative data of 5mC staining (**i**). Scale bar, 50 μm. Statistical significance was determined by unpaired two-tailed Student’s *t* test in (**c**, **e**, **f**). Statistical significance was determined by two-tailed Wilcoxon rank-sum test in (**i**). Experiments in (**a**) were repeated twice. Source data are provided as a Source Data file.

DNA methylation profiling in the TCGA cohort of PCa patients reveals a global DNA hypermethylation pattern in SPOP-mutated primary tumors^3^. Until now, however, it remains unclear whether SPOP mutations play any causal role in the formation of DNA-hypermethylated epigenome. The vast majority of SPOP mutations detected in PCa patients thus far are hemizygous missense mutations^3,14,15^. In agreement with the report that SPOP promotes protein degradation by forming a dimer or oligomer^12,33^, ectopically expressed SPOP mutant acts in a dominant-negative fashion to inhibit the activity of endogenous wild-type (WT) SPOP^17,34^. To recapitulate the scenario in patient samples, we stably expressed several PCa-derived SPOP mutants into 22Rv1 PCa cells and performed immunofluorescence cytochemistry (IFC) analysis of global DNA methylation using anti-5mC antibody which specifically recognizes methylated cytosine. We found that compared to the empty vector (EV), expression of SPOP mutants Y87C, F102C, F133V, and Q165P invariably increased DNA methylation in 22Rv1 cells and the differences are statistically significant (Fig. 1a–c). In contrast, ectopic expression of wild-type (WT) SPOP markedly decreased DNA methylation (Supplementary Fig. 1a, b), and re-introduction of WT SPOP largely reversed SPOP-mutant F133V-induced DNA hypermethylation, although the effect was not as rigorous as 5-azacytidine (5-AzaC), a DNMT inhibitor (Supplementary Fig. 1c–e). We extended our analysis using mouse embryonic fibroblasts (MEFs) generated from SPOP^LoxP-STOP-LoxP-*F102C*^ conditional mice as we reported previously^35^. We infected MEFs with lentivirus expressing CMV-driven Cre recombinase and found that transient induction of Myc-SPOP-F102C mutant significantly increased DNA methylation (Fig. 1d, e). We also performed immunohistochemistry (IHC) analysis with anti-5mC antibody in SPOP-mutant Q165P PCa patient-derived xenograft (PDX) tumors^14^. The 5mC signal intensity was higher in Q165P PDX tumors in comparison to SPOP-WT tumors (Fig. 1f, g). To examine the effect of SPOP mutations on 5mC levels in primary PCa patient specimens, we performed Sanger sequencing to detect SPOP mutations in a cohort of 84 cases of PCa in which we identified nine SPOP-mutated tumors (Supplementary Data 1). The SPOP mutation frequency in our samples (9/84, 10.71%) is consistent with previous findings in different PCa cohorts including TCGA^3,15^. Among the patient samples examined, approximately 90% of SPOP-mutated primary tumors exhibited strong or intermediate 5mC signals. In contrast, only 56% of SPOP-WT tumors had strong or intermediate 5mC signals (Fig. 1h, i, Supplementary Data 1). These findings are not only consistent with the detection of increased DNA methylation in SPOP-mutated PCa patient specimens^3,8^, but also provide direct evidence that SPOP mutations play a causal role in induction of DNA hypermethylation in PCa cells.

### GLP is a binding substrate of SPOP

In mammals, DNA methylation is directly regulated by three DNA methyltransferases including DNMT1, DNMT3A, and DNMT3B^36^. To define the molecular mechanism by which SPOP mutations drive DNA hypermethylation, we first examined their effect on DNMT protein expression. We found that the levels of DNMT1, DNMT3A, and DNMT3B proteins were comparable between SPOP WT and Q165P mutant PDX tumors (Supplementary Fig. 1f) although 5mC levels were much higher in Q165P PDX tumors (Fig. 1f, g). Corroborating this observation, we demonstrated that neither endogenous SPOP depletion by short hairpin RNAs (shRNAs) nor F102C and F133V mutant expression had any obvious effect on DNMT1, DNMT3A and DNMT3B protein expression in both 22Rv1 and DU145 PCa cell lines (Supplementary Fig. 1g, h). Co-immunoprecipitation (co-IP) assay indicated that ectopically expressed Myc-tagged SPOP failed to pull down endogenous DNMT proteins in 22Rv1 cells (Supplementary Fig. 1i). These results rule out the possibility that SPOP regulates DNA methylation by directly binding or modulating the expression of DNMT proteins in PCa cells.

**Fig. 2:**
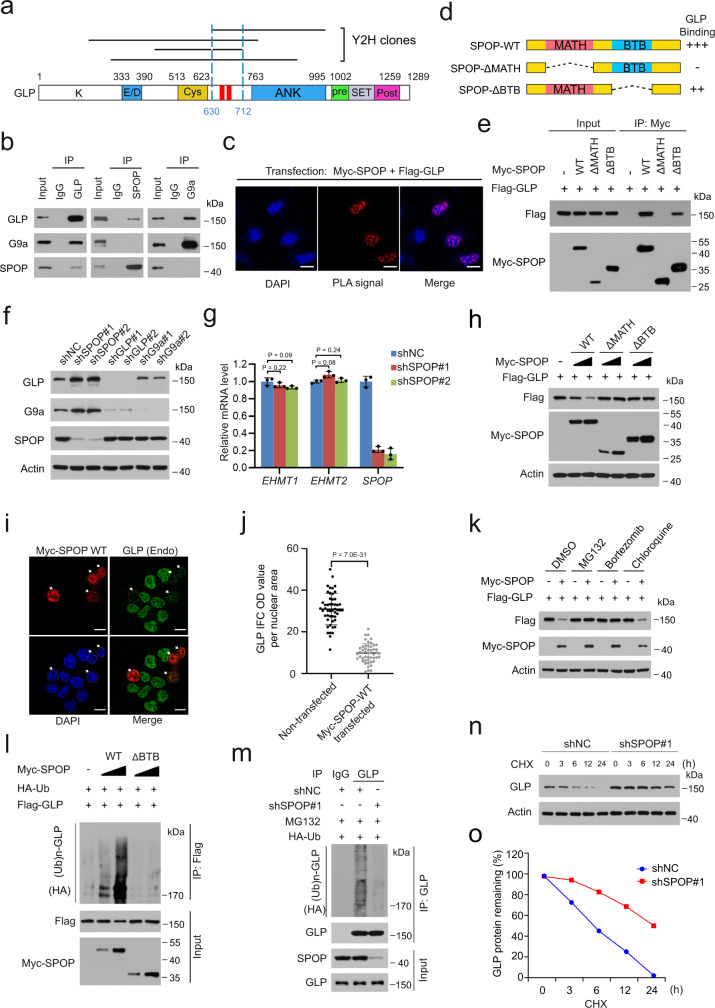
GLP is a ubiquitination and proteasomal degradation target of SPOP. **a** Diagram showing the four GLP clones identified from Y2H screen using full-length SPOP as bait. Minimal interacting region shared by positive clones (the region between two dashed blue lines) covers two putative SBC motifs (red rectangles). **b** Western blots of co-IP samples from 22Rv1 cells treated with 20 µM MG132 for 8 h. **c** Representative images of proximity ligation assay (PLA) in 22Rv1 cells transfected with indicated plasmids. Scale bar, 10 μm. **d** Schematic representation of SPOP deletion mutants indicating their binding capability with GLP. **e** Western blots of WCL and co-IP samples from 293T cells transfected with indicated plasmids and treated with 20 μM MG132 for 8 h. **f** Western blots of WCL from 22Rv1 cells expressing indicated shRNAs. **g** RT-qPCR analysis of indicated genes in 22Rv1 cells expressing indicated shRNAs. Data shown means ± SD (n = 3 replicates/group). **h** Western blots of WCL from 293T cells transfected with the indicated plasmids. **i**, **j** Representative IFC images of Myc-SPOP and GLP staining in 22Rv1 cells transfected with Myc-SPOP WT (**i**). Scale bar, 10 μm. GLP staining was quantified using ImageJ optical density (OD)/nuclear area (pixel) (**j**). Data shown means ± SD (*n* = 50 cells/group). **k** Western blots of WCL from 293T cells transfected with indicated plasmids and treated with DMSO, MG132 (20 μM), bortezomib (200 nM) or chloroquine (50 μM) for 8 h. **l** Western blots of WCL and co-IP samples from 293T cells transfected with the indicated plasmids and treated with 20 µM MG132 for 8 h. **m** Western blots of the products of in vivo ubiquitination assays from 22Rv1 cells expressing indicated shRNAs and HA-Ub. **n**–**o** 22Rv1 cells expressing indicated shRNA were treated with 50 μg/ml cycloheximide (CHX) and harvested at different time points for western blots (**n**). Quantification of GLP protein from western blots normalized to actin and then to 0-h time point (**o**). Statistical significance was determined by unpaired two-tailed Student’s *t* test in (**g**, **j**). Experiments in (**b**, **c**, **e**, **h**, **k**, **l**) were repeated twice. Source data are provided as a Source Data file.

To define in an unbiased manner the molecular mechanisms by which SPOP mutations regulate DNA methylation, we performed Y2H screen in a human fetal kidney cDNA library (preferably a library from prostate, but not available from the source—Takara/Clontech) using full-length SPOP as bait. While the majority of hits from this screen overlapped with the results of our previous screen performed in a brain cDNA library^17^ (e.g. DEK, BRD2/3/4, and GLI2/3), we identified a few new putative SPOP-interacting proteins including the histone H3K9 methyltransferase GLP (Fig. 2a). We chose to focus on GLP since it has been reported that GLP and its partner protein G9a are implicated in DNA methylation due to their binding of DNMT proteins^25,37,38^. Using co-IP assay, we confirmed that ectopically expressed SPOP interacted with GLP, but not G9a in 293T cells (Supplementary Fig. 2a). We found that GLP also interacted with SPOP and G9a (positive control) at the endogenous level in 22Rv1 cells; however, SPOP only interacts with endogenous GLP, but not G9a and vice versa in these cells (Fig. 2b). The interaction of SPOP and GLP was further confirmed by proximity ligation assay (PLA) in 22Rv1 cells with ectopic expression of Myc-SPOP and Flag-GLP (Fig. 2c). These data indicate that SPOP interacts with GLP in a complex independently of GLP/G9a complex in PCa cells.

SPOP contains two major structural domains, a substrate-binding MATH domain at the N-terminal portion and a CUL3-binding BTB domain at the C-terminal region. To determine which domain of SPOP mediates its interaction with GLP, we generated SPOP-ΔBTB and SPOP-ΔMATH mutants by deleting these domains individually (Fig. 2d). Co-IP assay in 293T cells showed that full-length SPOP (SPOP-WT) and SPOP-ΔBTB, but not SPOP-ΔMATH mutant efficiently interacted with GLP (Fig. 2e), suggesting that SPOP interacts with GLP through MATH domain.

### The CRL3^SPOP^ E3 ubiquitin ligase complex induces GLP proteasomal degradation

We examined whether GLP is a proteolytic substrate of SPOP. Knockdown (KD) of endogenous SPOP increased the steady-state level of endogenous GLP protein in 22Rv1, PC-3 and DU145 PCa cell lines (Fig. 2f, Supplementary Fig. 2b). SPOP KD also increased G9a protein levels (Fig. 2f, Supplementary Fig. 2b). This effect could be indirect since SPOP does not bind to G9a (Fig. 2b), but it has been shown that GLP is required for G9a stabilization^23^. Indeed, depletion of GLP largely decreased the G9a protein level in 22Rv1 cells (Fig. 2f). In contrast, SPOP KD had little or no effect on *EHMT1* and *EHMT2* mRNA expression (Fig. 2g), suggesting that SPOP regulates GLP expression at post-transcriptional level. Ectopic expression of SPOP WT decreased GLP protein expression in a dose-dependent manner, and this effect relies on both MATH (binding GLP) and BTB (binding CUL3 to be an active E3 ligase) domains of SPOP (Fig. 2h–j). However, SPOP-mediated decrease of GLP protein expression was completely blocked by the proteasome inhibitors MG132 or bortezomib, but not the lysosome inhibitor chloroquine (Fig. 2k), suggesting that SPOP promotes GLP proteasomal, but not the lysosomal degradation. MLN4924 is a small-molecule inhibitor of NEDD8-activating enzyme which is required for CRL complex activation^39^. Similar to the effect of MG132 and bortezomib, MLN4924 also caused GLP and G9a accumulation at the protein, but not mRNA level (Supplementary Fig. 2c, d). We also depleted CUL3 or RBX1, two key components of the CRL3^SPOP^ E3 ubiquitin ligase complex by two independent small interfering RNAs (siRNAs) in 22Rv1 cells. We found that CUL3 or RBX1 depletion substantially increased GLP and G9a protein levels (Supplementary Fig. 2e). We further showed that expression of WT SPOP augmented GLP polyubiquitination, but such effect was not observed in cells expressing the enzymatic dead ∆BTB mutant (Fig. 2l). Accordingly, depletion of endogenous SPOP decreased polyubiquitination of endogenous GLP protein (Fig. 2m). SPOP depletion also largely prolonged the half-life of endogenous GLP protein in 22Rv1 cells (Fig. 2n, o). Using both pharmacological and genetic approaches we demonstrate that the CRL3^SPOP^ E3 ubiquitin ligase complex promotes GLP polyubiquitination and proteasomal degradation in PCa cells.

### SPOP binds to two SBC motifs in GLP

Previous studies have shown that one or more SPOP-binding consensus (SBC) motifs are present in SPOP substrates^12^. We sought to determine whether GLP protein harbors any SBC motif. To this end, we focused on the minimal SPOP-interacting region in GLP (amino acids (a.a.) 630-712) defined by the four clusters of SPOP-binding-positive Y2H clones (Fig. 2a). Protein motif analysis in this region revealed that there are two perfectly matched SBC motifs that are very similar to the SBC motifs in known SPOP substrates such as MacroH2A, DAXX, DEK and BRD4^11,17,40,41^ (Supplementary Fig. 2f). To determine whether these putative motifs are required for SPOP-GLP interaction, we generated three GLP mutants SBC1-m, SBC2-m and SBC1/2-m, in which the serine and threonine residues in SBC motif were mutated to alanine individually or together (Supplementary Fig. 2g). Co-IP assay demonstrated that mutation of the first or second motif in GLP partially reduced its binding with SPOP; however, the double mutant SBC1/2-m completely abrogated its ability to bind SPOP (Supplementary Fig. 2h), indicating that both SBC motifs are required for SPOP-GLP interaction. Next, we examined whether both motifs are required for SPOP-mediated GLP degradation. GLP-SBC1-m and GLP-SBC2-m mutants were still degraded by SPOP, albeit not as effectively as the WT counterpart (Supplementary Fig. 2i). By contrast, the double mutant GLP-SBC1/2-m was completely resistant to SPOP-mediated degradation (Supplementary Fig. 2i). Polyubiquitination assays were performed to further determine the importance of these two motifs as degrons. Deletion of these two motifs totally abolished SPOP-mediated GLP polyubiquitination (Supplementary Fig. 2j). Moreover, the SBC1/2-m double mutations significantly prolonged GLP protein half-life (Supplementary Fig. 2k, l). These data reveal two functional SBC motifs that are essential for SPOP binding and polyubiquitination-dependent degradation of GLP.

### SPOP mutation increases GLP and G9a protein level in PCa cell lines, PDX tumors and patient specimens

**Fig. 3:**
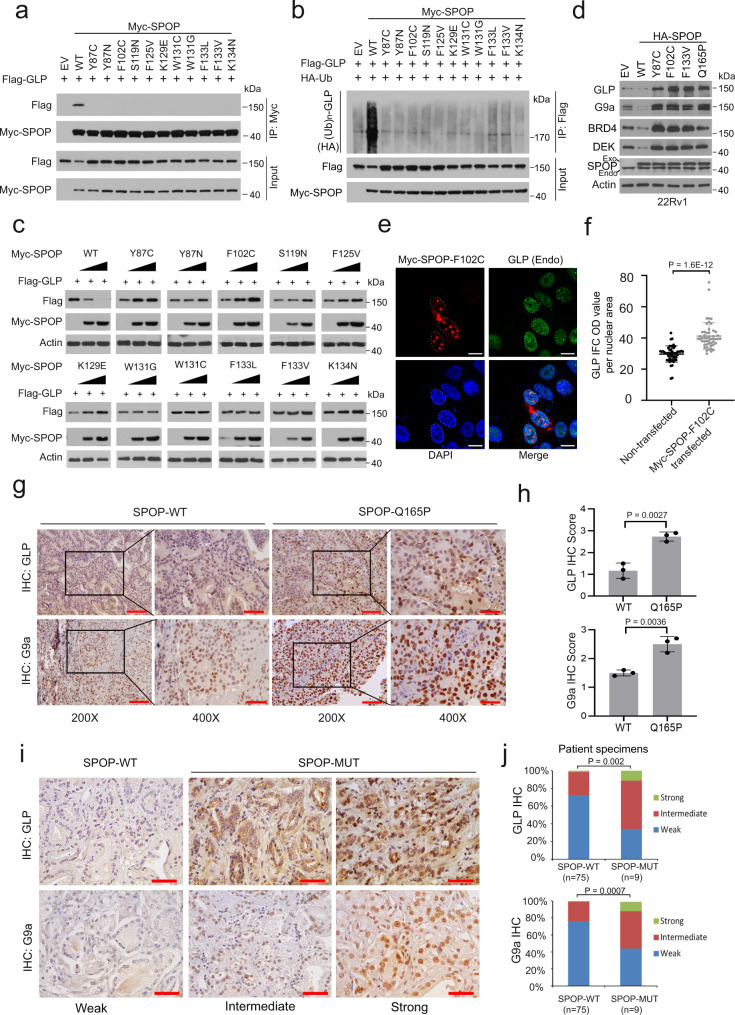
GLP/G9a protein is elevated in SPOP-mutant PCa cells in culture and in patient specimens. **a** Western blots with indicated antibodies in WCL and co-IP samples from 293T cells transfected with plasmids of empty vector (EV) or indicated SPOP mutants and treated with 20 µM MG132 for 8 h. **b** Western blots with indicated antibodies in WCL and co-IP samples of anti-Flag antibody from 293T cells transfected with the indicated plasmids and treated with 20 µM MG132 for 8 h. **c** Western blots of WCL from 293T cells transfected with the indicated plasmids. **d** Western blots of WCL from 22Rv1 cells stably infected with lentivirus expressing EV, WT or mutant HA-SPOP. **e**, **f** Representative IFC images of Myc-SPOP and endogenous GLP staining in 22Rv1 cells transfected with Myc-SPOP F102C (**e**) and the optical density (OD)/nuclear area (pixel) of GLP staining was quantified using ImageJ (**f**). Data shown means ± SD (*n* = 50 cells/group). Scale bar, 10 μm. **g**, **h** Representative IHC images of GLP and G9a staining in SPOP-WT and Q165P mutant PDX tumors (**g**) and the quantitative data of GLP and G9a staining (**h**). Scale bar in 200X images, 100 μm; Scale bar in 400X images, 50 μm. Data shown means ± SD (*n* = 3 replicates/group). **i**, **j** Representative IHC images of GLP and G9a staining in 84 PCa patient specimens (**i**) and the quantitative data of GLP and G9a staining (**j**). Scale bar, 50 μm. Statistical significance was determined by unpaired two-tailed Student’s *t* test in (**f**, **h**). Statistical significance was determined by two-tailed Wilcoxon rank-sum test in (**j**). Experiments in (**a**–**d**) were repeated twice. Source data are provided as a Source Data file.

The vast majority of PCa-associated SPOP mutations mainly occur in the MATH domain^14^, which binds to targeting substrates. We postulated that PCa-associated mutants of SPOP may be defective in mediating GLP protein destruction. We performed co-IP assays and demonstrated that different from WT SPOP, all PCa-associated SPOP mutants we examined failed to bind to GLP (Fig. 3a). SPOP-mediated degradation and ubiquitination of GLP protein were also impaired by these mutations (Fig. 3b, c). Accordingly, stable expression of a few well-characterized PCa-associated SPOP mutants including Y87C, F102C, F133V and Q165P^14^ increased the protein level of endogenous GLP/G9a in both 22Rv1 and DU145 cells (Fig. 3d, Supplementary Fig. 3a). We also performed IFC analysis and showed that endogenous GLP and G9a protein levels were much higher in 22Rv1 cells transiently transfected with SPOP-F102C mutant compared to non-transfected control cells (Fig. 3e, f, Supplementary Fig. 3b, c). In contrast, no such effect was observed on mRNA expression of *EHMT1* and *EHMT2* genes in these two cell lines, and similar results were observed in three additional PCa cell lines including LNCaP, C4-2 and PC-3 (Supplementary Fig. 3d, e).

We further examined the expression of GLP and G9a proteins in PDX tumors and patient samples by IHC. We found that their expression was upregulated in SPOP Q165P mutant PDXs and SPOP-mutated PCa patient samples compared to SPOP WT counterparts (Fig. 3g–j, Supplementary Data 1). Together, these data demonstrate that both GLP and G9a protein levels are elevated in SPOP-mutated PCa cells in culture, PDX tumors and patient specimens.

### SPOP mutation induces global DNA hypermethylation via GLP/G9a

**Fig. 4:**
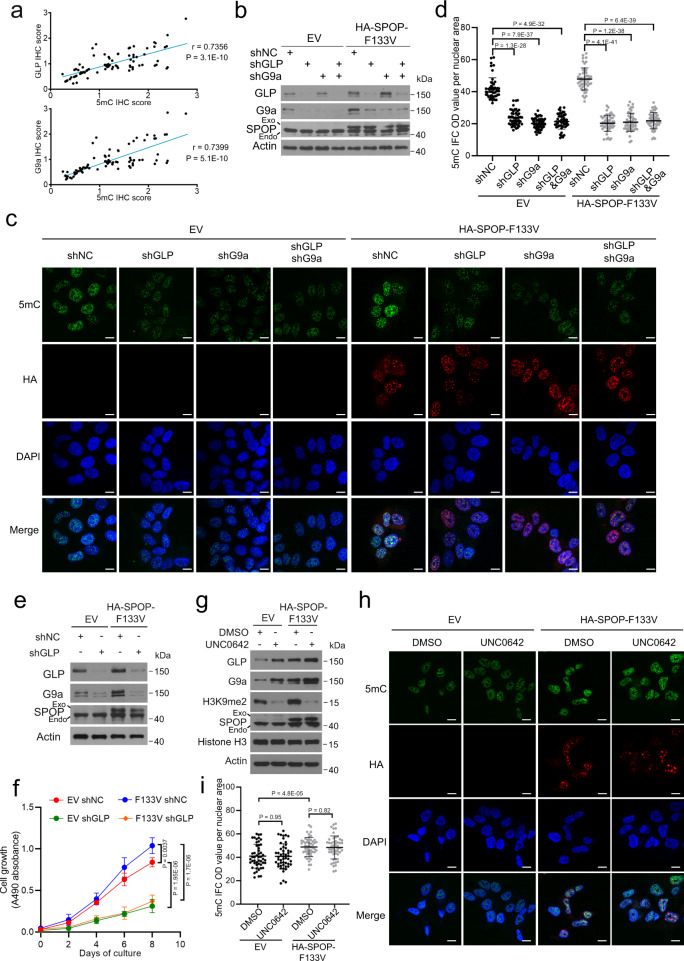
GLP/G9a mediate SPOP mutation-induced hypermethylation independent of its HMTase activity. **a** Pearson correlation of 5mC and GLP/G9a IHC scores in 84 PCa patient specimens. **b**–**d** Western blots of WCL from 22Rv1 cells infected with lentivirus expressing indicated constructs and shRNAs (**b**). Representative IFC images of 5mC and HA-SPOP F133V staining are shown in (**c**), and ImageJ was used to quantify the optical density (OD)/nuclear area (pixel) of 5mC signals (**d**). Data shown means ± SD (*n* = 50 cells/group). Scale bar, 10 μm. **e**, **f** 22Rv1 cells infected with lentivirus expressing indicated constructs and shRNAs were harvested for Western blots with indicated antibodies (**e**) or used for MTS assay to measure cell proliferation (**f**). Data shown means ± SD (*n* = 5 replicates/group). **g**-**i** 22Rv1 cells infected with lentivirus expressing EV and SPOP F133V and treated with 2 µM UNC0642 for 24 h for western blots (**g**). Representative IFC images of 5mC and HA-SPOP F133V staining are shown in (**h**) and 5mC signals in each group were quantified using ImageJ (**i**). Data shown means ± SD (*n* = 50 cells/group). Scale bar, 10 μm. Statistical significance was determined by unpaired two-tailed Student’s *t* test in (**a**, **d**, **f**, **i**). Experiments in (**b**, **e**, **g**) were repeated twice. Source data are provided as a Source Data file.

In addition to the finding that both global DNA methylation and GLP/G9a protein expression were much higher in SPOP-mutated patient samples compared to SPOP WT counterparts (Figs. 1h, i, 3i, j, Supplementary Data 1), we further showed that high GLP/G9a expression also correlated with DNA hypermethylation in patient specimens (Fig. 4a). These results prompted us to determine whether upregulation of GLP/G9a protein plays a causal role in SPOP mutation-induced increase in DNA methylation. To this end, we knocked down GLP, G9a or together in control (expressing EV) and SPOP F133V stable 22Rv1 cells. We performed 5mC IFC analysis and demonstrated that knockdown of GLP or G9a individually or together not only decreased 5mC level in control cells, but also completely abolished SPOP F133V-induced increase in 5mC level (Fig. 4b–d). In contrast, knockdown of a few other known degradation substrates of SPOP including AR, BRD4 and PD-L1^16,17,19,21^ did not result in any obvious changes in global DNA methylation in either EV or SPOP-F133V-expressing cells (Supplementary Fig. 4a–c). Moreover, knockdown of GLP (G9a concomitantly downregulated) blocked SPOP F133V-enhanced proliferation of 22Rv1 cells (Fig. 4e, f). Besides other functions such as binding to DNMT proteins, the GLP/G9a complex primarily acts as a HMTase to catalyze methylation of H3K9 and non-histone proteins^42^. Indeed, while stable expression of SPOP F133V mutant increased protein expression of both GLP and G9a, it also elevated H3K9me2 level in 22Rv1 cell (Fig. 4g). As expected, treatment of cells with the GLP/G9a methyltransferase inhibitor UNC0642 inhibited H3K9me2 in both control and SPOP F133V-expressing 22Rv1 cells (Fig. 4g). However, UNC0642 treatment did not affect global DNA methylation and cell proliferation regardless of F133V status (Fig. 4g–i, Supplementary Fig. 4d). These findings indicate that GLP/G9a play a key role in mediating SPOP mutation-induced DNA hypermethylation and proliferation in PCa cells and these effects are independent of the H3K9me2 methyltransferase activity, consistent with an HMTase-independent role of GLP/G9a in regulating DNA methylation reported previously^24,43^. Notably, the enzymatic function of GLP/G9a is important for SPOP mutation-enhanced cell migration (Supplementary Fig. 4e–i).

### SPOP mutations induce TSG methylation via elevated GLP and G9a proteins

**Fig. 5:**
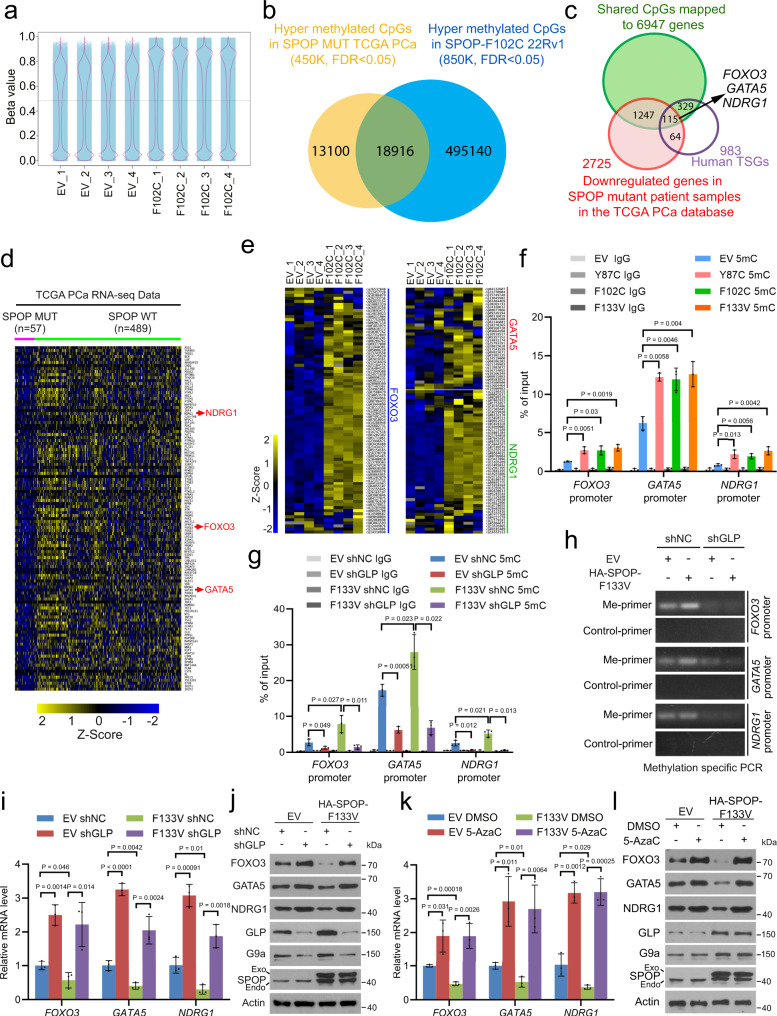
SPOP-GLP axis regulates DNA methylation and TSG expression. **a** A Jitter plot comparison of overall beta value in 22Rv1 cells expressing empty vector (EV) or SPOP F102C mutant with four replicates per group. **b** Venn diagram showing the overlap between hypermethylated CpGs from EPIC 850K array (F102C vs EV) in 22Rv1 cells (*n* = 514056, FDR < 0.05) and hypermethylated CpGs from 450K (SPOP mutated vs WT) in the TCGA database (*n* = 32016, FDR < 0.05). Two-tailed Hypergeometric test, *P* = 1.07E−07. **c** Venn diagram showing the overlap among genes with increased DNA methylation in SPOP F102C expressing 22Rv1 (EPIC 850K) and SPOP mutant patient sample (450K), genes downregulated in SPOP-mutated prostate tumors in the TCGA cohort, and tumor suppressor genes reported previously (https://bioinfo.uth.edu/TSGene/index.html?csrt=17897587749110957416). Two-tailed Hypergeometric test, *P* = 9.01E−05. **d** Heat map shows the differential expression of 115 overlapped genes identified in (**c**) in SPOP-mutated compared to SPOP WT tumors in the TCGA cohort. **e** Heat map shows the methylation alterations in the CpGs mapped to *FOXO3*, *GATA5* and *NDRG1* gene loci in F102C-expressing compared to EV control 22Rv1 cells. **f** MeDIP-qPCR analysis of DNA methylation in the indicated gene promoter in EV or SPOP mutant expressing 22Rv1 cells. Data shown means ± SD (*n* = 3 replicates/group). **g**, **h** Analysis of DNA methylation in the indicated gene promoters in 22Rv1 cells infected with lentivirus expressing indicated plasmids and shRNAs using MeDIP-qPCR (**g**) and methylation-specific PCR (**h**). Data shown means ± SD (*n* = 3 replicates/group). **i**, **j** Measurement of *FOXO3*, *GATA5*, *NDRG1* mRNA and protein expression in 22Rv1 cells expressing indicated plasmids and shRNAs using RT-qPCR (**i**) and western blot (**j**). Data shown means ± SD (*n* = 3 replicates/group). **k**, **l** Analysis of *FOXO3*, *GATA5* and *NDRG1* mRNA and protein expression by RT-qPCR (**k**) and western blot (**l**), respectively in EV and F133V-expressing 22Rv1 cells treated with or without 2 μM 5-AzaC for 48 h. Data shown means ± SD (*n* = 3 replicates/group). Statistical significance was determined by unpaired two-tailed Student’s *t* test in (**f**, **g**, **i**, **k**). Experiments in (**j**, **l**) were repeated twice. Source data are provided as a Source Data file.

To globally define methylated genomic loci induced by SPOP mutation in PCa cells, we performed DNA methylation analysis using Illumina Infinium MethylationEPIC BeadChip (850K) in control (EV) and SPOP F102C-expressing 22Rv1 cells. The data from the principal component analysis (PCA) plot, scatter plots and density plot show that the overall agreement is excellent among the biological replicates of both control and F102C-expressing cells (Supplementary Fig. 5a–c, Supplementary Data 2–4). We found that the DNA methylation level in SPOP F102C-expressing cells was much higher compared to EV cells (Fig. 5a, Supplementary Fig. 5c, d), consistent with the IFC data showing that SPOP mutation induces an overall increase in DNA hypermethylation (Fig. 1). We demonstrated that the differentially methylated CpGs are located in CpG islands (CGIs), promoters, exons, introns and intergenic regions (Supplementary Fig. 5e). We identified 514,056 CpGs (FDR < 0.05) with methylation level much higher in F102C-expressing 22Rv1 cells compared to EV control cells (Fig. 5b, Supplementary Data 5). By clustering our MethylationEPIC BeadChip data with the TCGA 450K array data in patients (32016 CpGs with increased methylation in SPOP-mutant samples compared to SPOP WT counterparts) (Fig. 5b, Supplementary Data 6), we identified 18,916 shared hypermethylated CpGs, which are mapped to 6,947 genes (Fig. 5b, c, Supplementary Data 7, 8). Among the 6,947 commonly methylated genes, 1,362 genes are overlapped with 2725 genes downregulated in SPOP-mutated TCGA patient samples and among them, 115 genes are known tumor suppressor genes (Fig. 5c, d, Supplementary Data 9).

Pathway analysis of the 115 hypermethylated downregulated TSGs indicates that these genes are enriched in several cancer-relevant pathways including hematopoietic stem cell gene regulation, VEGFA-VEGFR signaling pathway and adenoid cystic carcinoma (Supplementary Fig. 5f). Among them, some are well-characterized TSGs including *FOXO3*, *GATA5,* and *NDRG1* and importantly, these genes were highly methylated in both cultured SPOP-mutant 22Rv1 cells and TCGA patient samples (Fig. 5e, Supplementary Fig. 5g). SPOP mutation-enhanced DNA methylation in these gene loci was further validated by the independent methylated DNA immunoprecipitation quantitative PCR (MeDIP-qRCR) analysis in the promoter region of these TSGs (Fig. 5f). Moreover, meta-analysis showed a similar trend of DNA methylation in *FOXO3*, *GATA5,* and *NDRG1* gene loci in a cohort of metastatic castration-resistant prostate cancer (mCRPC) patient samples^7^ (Supplementary Fig. 5h–j). Methylation of *NDRG1* gene also negatively correlated with its expression in the TCGA patient samples (Supplementary Fig. 5k). In addition, increased methylation of these genes in SPOP-mutant cells is reversed by GLP knockdown (Fig. 5g, h). In contrast, knockdown of several other SPOP degradation substrates including AR, BRD4, and PD-L1 did not reverse SPOP F133V-induced hypermethylation of these genes (Supplementary Fig. 6a, b). Furthermore, re-introduction of WT SPOP or treatment of the DNA demethylation reagent 5-AzaC in SPOP F133V-expressing 22Rv1 cells effectively reversed SPOP mutation-induced hypermethylation at these gene loci (Supplementary Fig. 6c). SPOP mutation-induced hypermethylation of representative genes in other five affected pathways was also confirmed by MeDIP-qRCR in 22Rv1 cells (Supplementary Fig. 6d–h).

Real-time quantitative reverse transcription PCR (RT-qPCR) and western blot analyses revealed that expression of *FOXO3*, *GATA5* and *NDRG1* genes were suppressed by SPOP F133V at both mRNA and protein levels in 22Rv1 cells, and this effect was reversed by GLP depletion or 5-AzaC treatment, but not affected by knockdown of other SPOP substrates such as AR, BRD4 and PD-L1 (Fig. 5i–l, Supplementary Fig. 6a). Similar to the effects of SPOP mutations, SPOP knockdown also decreased expression of these TSG proteins while increasing GLP and G9a level (Supplementary Fig. 7a). Increased level of GLP and G9a and decreased expression of these targets were observed in SPOP Q165P PDX tumors compared to control tumors (Supplementary Fig. 7b). Moreover, we demonstrated that deregulation of the SPOP-GLP axis also suppressed expression of these TSGs via DNA methylation in another cell line DU145 (Supplementary Fig. 7c–f). These data indicate that SPOP mutations induce TSG promoter hypermethylation and their downregulation via stabilization of GLP/G9a in PCa cells.

While UNC0642 treatment of 22Rv1 cells reduced H3K9me2 level, it failed to reverse F133V-induced repression of *FOXO3*, *GATA5,* and *NDRG1* gene expression at both protein and mRNA levels although as expected, UNC0642 blocked repression of *LATS2*, a gene reportedly regulated by the GLP/G9a HMTase activity^44^ in F133V-expressing cells (Supplementary Fig. 7g, h). We further showed that UNC0642 treatment was also unable to reverse F133V-induced upregulation of DNA methylation in the promoter of *FOXO3*, *GATA5* and *NDRG1* genes (Supplementary Fig. 7i). Thus, consistent with the effect on global DNA methylation (Fig. 4), these results suggest that the impact of GLP/G9a on DNA methylation, at least in a subset of TSG promoters is unlikely mediated through their HMTase activity in SPOP-mutated PCa cells.

### SPOP mutations regulate DNA methylation and TSG gene expression via GLP/G9a interaction with DNMTs

**Fig. 6:**
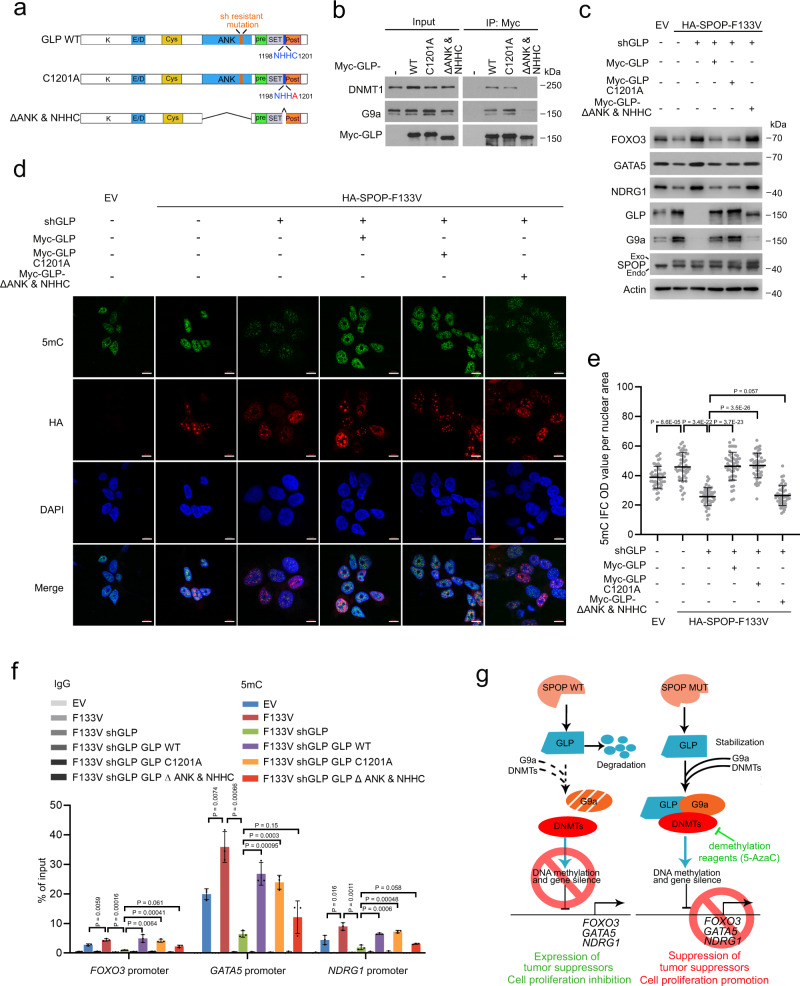
SPOP mutation regulates DNA methylation through GLP/G9a interaction with DNMT proteins. **a** Diagram showing wild-type GLP, enzymatic inactive mutant C1201A and ANK & NHHC domain double deletion mutant. **b** Western blots with indicated antibodies in WCL and co-IP samples from 22Rv1 cells transfected with indicated plasmids. **c**–**f** 22Rv1 cells infected with lentivirus expressing indicated plasmids and/or shRNAs and transfected with indicated constructs were used for western blot (**c**), IFC (**d**) and MeDIP-qPCR (**f**). ImageJ was used to quantify the optical density (OD)/nuclear area (pixel) of 5mC staining in each group. Data shown means ± SD (*n* = 50 cells/group) (**e**). Scale bar, 10 μm. Data shown means ± SD (*n* = 3 replicates/group) (**f**). **g** A working model based on the current findings. **Left**, WT SPOP recognizes and promotes proteasomal degradation of GLP, thereby destabilizing the GLP/G9a complex, inhibiting GLP/G9a-mediated interaction with DNMTs and DNA methylation, and inducing expression of tumor suppressors such as FOXO3 and inhibition of cell proliferation. **Right**, PCa-associated SPOP mutations fail to bind and degrade GLP, thereby inducing elevation of the GLP/G9a complex and their interaction with DNMTs, increasing DNA methylation and suppression of expression of tumor suppressors and promoting cell proliferation. However, this process can be reversed by DNA demethylation reagents such as 5-AzaC in SPOP-mutant cells. Statistical significance was determined by unpaired two-tailed Student’s *t* test in (**e**, **f**). Experiments in (**b**, **c**) were repeated twice. Source data are provided as a Source Data file.

The ANK domains in both GLP and G9a are important for DNMT binding and the NHHC domain in GLP is essential for G9a binding^24,25,38^. To determine the importance of the DMNT binding function of GLP/G9a in mediating DNA hypermethylation in SPOP-mutant cells, we generated GLP DNMT-binding deficient mutant ΔANK&NHHC (cannot bind to DNMT proteins directly due to lack of ANK domain and indirectly due to loss of NHHC domain, which binds to G9a) and the enzymatically inactive mutant C1201A (negative control) (Fig. 6a). Co-IP analysis showed that C1201A mutant bound to DNMT1 to an extent similar to WT GLP, but ΔANK&NHHC mutant was unable to interact with DNMT1 in 22Rv1 cells (Fig. 6b). As expected, the binding of ΔANK&NHHC mutant to G9a was abolished (Fig. 6b). IFC and dot blot showed that SPOP mutation-induced hypermethylation was diminished by GLP knockdown and rescued by shRNA-resistant WT or enzymatically inactive C1201A mutant but not ΔANK&NHHC mutant (Fig. 6c–e, Supplementary Fig. 8a). Similarly, MeDIP-qPCR analysis demonstrated that GLP knockdown abolished F133V-induced DNA hypermethylation at *FOXO3*, *GATA5,* and *NDRG1* gene promoters and this effect was reversed by restored expression of shRNA-resistant GLP WT or enzymatically inactive C1201A but not ΔANK&NHHC mutant (Fig. 6f). Consistent with these results, both RT-qPCR and WB analyses showed that ectopic expression of shRNA-resistant GLP WT or C1201A mutant but not ΔANK&NHHC deletion mutant reversed the upregulation of these TSGs in GLP-depleted 22Rv1 cells (Fig. 6c, Supplementary Fig. 8b). In addition, cell proliferation can also be rescued by GLP WT or C1201A mutant but not ΔANK&NHHC deletion mutant (Supplementary Fig. 8c). These data further stress the importance of ANK&NHHC domains of GLP and the recruitment of DNMTs in mediating SPOP mutation-enhanced DNA methylation. Expression of SPOP WT or SPOP mutant did not affect GLP dimerizing with G9a and binding of DNMT1 when protein degradation was blocked by MG132 (Supplementary Fig. 8d, e), suggesting that SPOP does not affect GLP binding affinity with G9a or DNMT1. Moreover, forced expression of FOXO3 or NDRG1 individually partially reversed SPOP F133V expression-enhanced growth of DU145 cells (Supplementary Fig. 9a–d), suggesting that decreased expression of these TSGs due to promoter methylation contributes to SPOP mutation-augmented PCa cell growth. Collectively, these results support the notion that DNMT binding, but not the HMTase activity of the GLP/G9a complex is essential for SPOP mutation-induced global DNA methylation and epigenetic silencing of these TSGs (Fig. 6g).

### DNA methylation inhibitor sensitizes SPOP-mutant PCa cells to taxane in vitro and in vivo

**Fig. 7:**
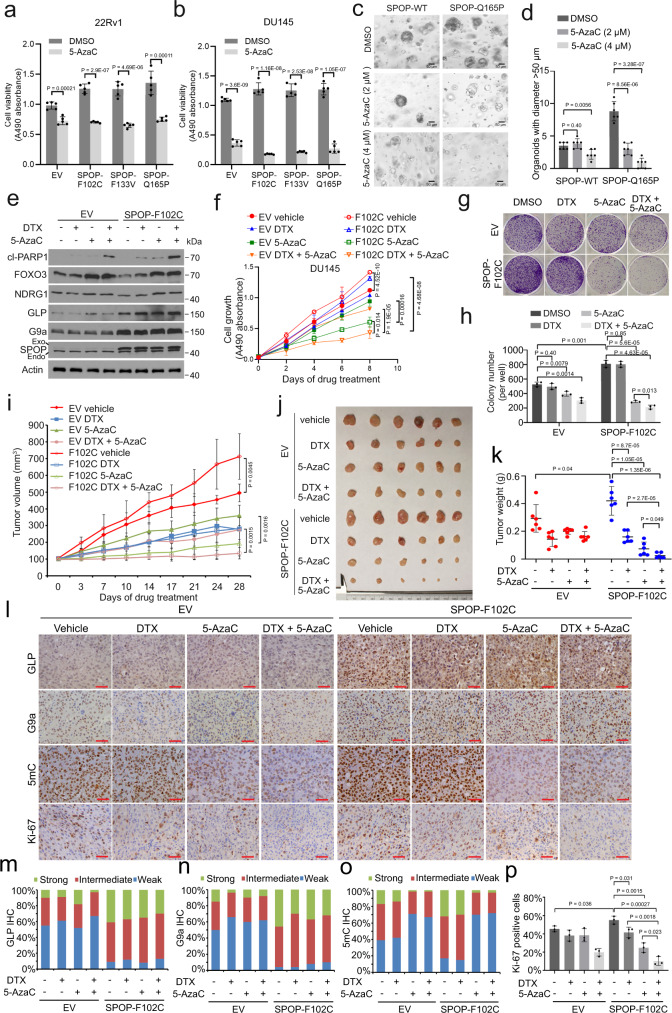
SPOP-mutant PCa cells are sensitive to DNA demethylation agent alone or in combination with taxane. **a**, **b** Cell viability was measured by MTS assay in 22Rv1 (**a**) and DU145 cells (**b**) expressing indicated constructs and treated with 5-AzaC (2 μM) for 5 days. Data shown means ± SD (*n* = 5 replicates/group). **c**, **d** Organoids derived from indicated PDX tumors were cultured with matrigel and treated with DMSO or 5-AzaC (2 or 4 μM) for 7 days followed by photography (**c**) and quantification (**d**). Scale bars, 50 μm. Data shown means ± SD (*n* = 6 fields/group). **e**, **f** DU145 cells expressing EV or SPOP-F102C were treated with DMSO, DTX (0.05 nM), 5-AzaC (2 μM) or both for 48 h and harvested for Western blots (**e**) or subjected to MTS assay (**f**). Data shown means ± SD (*n* = 5 replicates/group). **g**, **h** DU145 cells expressing EV or SPOP-F102C and treated with drugs as in (**e**) were subjected to colony formation assay for 12 days followed by photographing (**g**) and quantification (**h**). Data shown means ± SD (*n* = 3 replicates/group). **i**–**k** DU145 cells expressing EV or SPOP-F102C were injected s.c. into SCID male mice and treated with vehicle, DTX (5 mg/kg), 5-AzaC (2 mg/kg) or the combination of DTX and 5-AzaC. Tumor volume was measured at indicated time points (**i**). Tumors were harvested at day-28 and photographed (**j**), and tumor weight was measured (**k**). Data shown means ± SD (*n* = 6 replicates/group). **l** IHC staining for GLP, G9a, 5mC and Ki-67 was performed. Representative images were taken from each group. Experiments were repeated twice. Scale bar, 50 μm. **m**–**p** Quantification of IHC data shown in (**l**). Percentage of the cells with different intensity of staining (weak, intermediate and strong) for GLP (**m**), G9a (**n**) and 5mC (**o**) were determined. Percentage of Ki-67 staining-positive cells was quantified in (**p**). Data shown means ± SD (*n* = 3 xenograft tissues/group). Statistical significance was determined by unpaired two-tailed Student’s *t* test in (**a**, **b**, **d**, **f**, **h**, **i**, **k**, **p**). Experiments in (**e**) were repeated twice. Source data are provided as a Source Data file.

The DNA demethylation agent 5-AzaC has been approved for the treatment of malignancies with aberrant DNA methylation such as myelodysplastic syndrome (MDS) and other types of leukemia^45,46^. A combination of 5-AzaC with other agents such as HDAC inhibitors has been shown to be effective in certain cancer types^47,48^. Our mechanistic model (Fig. 6g) predicts that reversing SPOP mutation-mediated DNA hypermethylation and epigenetic silencing of TSGs by DNA demethylation agents should effectively restore TSG expression and inhibit SPOP mutation-accelerated PCa cell growth. Since SPOP mutations occurred mostly in PTEN-positive PCa, we examined the effect of SPOP mutation in two PTEN-positive PCa cell lines 22Rv1 and DU145. As expected, the expression of SPOP mutants in 22Rv1 and DU145 enhanced cell growth, but this growth advance was blocked by 5-AzaC treatment (Fig. 7a, b). We generated organoids from SPOP WT and Q165P mutant PCa PDXs^14^. 5-AzaC treatment also blocked Q165P mutant-enhanced growth of organoids in 3D culture (Fig. 7c, d). Thus, the inhibition effect of 5-AzaC was more prominent in SPOP-mutant cells and organoids compared to SPOP WT controls, indicating that DNA demethylation agent can inhibit SPOP-mutated PCa cell growth in vitro.

Taxane including docetaxel (DTX) is a first-line chemotherapy approved for treatment of high risk or metastatic PCa. While DTX and its natural analog paclitaxel (PTX) are known to cause cell cycle arrest by binding to and stabilize microtubules, they also induce apoptosis by causing nuclear localization and transactivation of FOXO family proteins such as FOXO3 in certain cancer cell types^49–51^. As expected, we found that DTX treatment enhanced FOXO3 nuclear localization of in 22Rv1 cells (Supplementary Fig. 9e). Therefore, we sought to test the hypothesis that co-treatment of 5-AzaC (increasing expression level of FOXO3 and other TSG proteins) sensitizes SPOP-mutated PCa cells to DTX (causing FOXO3 nuclear localization). As expected, 5-AzaC administration increases expression of FOXO3 and NDRG1 proteins in SPOP F102C-expressing DU145 cells in the presence or absence of DTX treatment (Fig. 7e). Importantly, both MTS and colony formation assays demonstrated that 5-AzaC treatment not only effectively inhibited the growth of SPOP F102C-expressing DU145 cells, but also drastically sensitized these cells to DTX (Fig. 7e–h). The noticeable anti-cancer effects of 5-AzaC alone or in combination with DTX were reminiscent of their effect on expression of cleaved PARP, an indicator of apoptotic cell death (Fig. 7e), suggesting that these effects were mediated, at least partially through induction of apoptosis. Similar results were obtained from DU145 xenograft studies (Fig. 7i–k). IHC analyses showed that expression of GLP, G9a and 5mC were higher in SPOP-F102C xenografts but as expected, the level of 5mC, but not GLP or G9a was reversed by 5-AzaC treatment (Fig. 7l–o). In agreement with these observations, Ki-67 expression was much higher in F102C mutant tumors compared to control counterparts, but this effect was abolished by 5-AzaC treatment alone or in combination with DTX (Fig. 7l, p). Taken together, these results suggest that combination of demethylation reagent with taxane represents a viable therapeutic strategy for the effective treatment of SPOP-mutated PCa.

## Discussion

Apart from other genetic subtypes, SPOP-mutated primary PCa possess a few prominent pathobiological features such as DNA hypermethylation^3,8^. Our data rule out the possibility that SPOP modulates DNA methylation through regulating expression and/or direct binding of DNMT proteins. Rather, we identify the HMTase GLP as a novel degradation substrate of SPOP and show that GLP protein and its partner G9a are highly elevated in cultured SPOP-mutated PCa cells and patient samples. Importantly, we reveal that the ability to bind DNMT proteins, but not the HMTase enzymatic activity is essential for the GLP/G9a complex to mediate SPOP mutation-induced DNA hypermethylation. Thus, our study reveals the aberrant elevation of GLP/G9a complex and their binding of DNMT proteins as a key mechanism to mediate the DNA-hypermethylated epigenome in SPOP-mutated PCa (Fig. 6g, **right**).

Molecular subtyping of cancer provides a significant advantage for precision medicine. In agreement with the observation that SPOP-mutated tumors adapt a DNA-hypermethylated epigenome, we demonstrate that 5-AzaC treatment effectively reverses DNA methylation-mediated silencing of TSGs, supporting the use of 5-AzaC for the treatment of SPOP-mutated subtype of this disease (Fig. 6g, **right**). Clinically, 5-AzaC has not been approved for PCa therapy and phase I/II clinical trials with the combination of 5-AzaC and DTX has been conducted for PCa patients. Although the combined therapy outperforms 5-AzaC alone, the objective response rate was relatively small^52^, highlighting that certain biomarkers are necessary for the combined therapy. We demonstrate that while 5-AzaC can effectively inhibit SPOP-mutant PCa cell growth, it further enhances the anti-cancer efficacy of DTX both in vitro and in mice. Thus, SPOP mutations could serve as a biomarker to guide the effective treatment of PCa with DNA demethylation agent alone or in combination with the first-line chemotherapeutics taxane.

## Methods

### Antibodies and chemicals

The antibodies and chemicals used in this study are summarized in Supplementary Data 10.

### Plasmids and mutagenesis

Expression vectors for WT SPOP and mutants were generated in our lab. Flag-GLP and Flag-G9a plasmids were kind gifts from Dr. Xiaochun Yu (Beckman Research Institute, City of Hope). GLP mutants were generated with KOD Plus mutagenesis Kit (Toyobo) following the manufacturer’s instructions. Primers used to generate GLP mutants are listed in Supplementary Data 11.

### Cell culture, transfection, and lentivirus infection

PC-3, 22Rv1, DU145, and 293T cells were obtained from the American Type Culture Collection (ATCC). The 293T cells were maintained in DMEM supplemented with 10% FBS, and PC-3, DU145 and 22Rv1 cells were maintained in RPMI medium supplemented with 10% FBS. For transient transfection, cells were transfected with PEI (Polyethyleneimine) or Lipofectamine RNAi MAX (for siRNA transfection) (Thermo Fisher Scientific) according to the manufacturer’s instructions. For lentivirus transfection, the pTsin-SPOP-F102C or F133V mutant expression vector or pLKO-base gene shRNA knockdown plasmids were transfected into 293T cells. Virus-containing supernatant was harvested 48 h after transfection to infect 22Rv1 and DU145 cells with 10 μg/ml polybrene. The successfully infected cells were selected with 1 μg/ml puromycin. ShRNA plasmids were purchased from Sigma-Aldrich. The shRNA sequences targeting SPOP, GLP or G9a are listed in Supplementary Data 12.

### Yeast two-hybrid screen assays

The yeast two-hybrid screen was performed with full-length SPOP cloned in frame with the GAL4 DNA-binding domain in vector PGBKT7 (Takara - Clontech). Yeast cells were transformed with PGBKT7-SPOP and a human fetal kidney cDNA library (Takara - Clontech). A total of 2 × 10^7^ independent clones were screened by growth in deficient medium and X-gal staining. Positive clones were subsequently retested in fresh yeast cells, and the identities of prey were determined with interaction sequence tags (ISTs) obtained by DNA sequencing.

### RNA interference

Nonspecific control siRNA and gene-specific siRNAs for human RBX1 and CUL3 were purchased from Thermo Fisher Scientific Dharmacon. SiRNA transfection of cells was performed following the manufacturer’s instructions. The sequences of the siRNA oligonucleotides are listed in Supplementary Data 12.

### Co-immunoprecipitation (co-IP)

Immunoprecipitation were performed as described previously^53^. For co-IP of ectopically expressed proteins, cells transfected with indicated plasmids were harvested and lysed by lysis buffer (50 mM Tris-HCl pH 7.5, 150 mM NaCl, 0.5% Nonidet P-40, and freshly added protease inhibitor cocktails). The whole-cell lysate was centrifuged and the supernatant was used to incubate with indicated antibody and protein A/G beads at 4 °C overnight. The beads were washed 3 times using lysis buffer. The samples were heated in SDS loading buffer (Thermo Fisher Scientific) for western blot. For IP of endogenous proteins, cells were lysed with lysis buffer and centrifuged to obtain supernatant. Protein A/G beads and indicated antibody were used to incubate with the supernatant at 4 °C overnight. Beads were washed 3 times with lysis buffer, re-suspended in SDS loading buffer prior to western blot analysis.

### Western blot

Whole-cell lysates or IP samples were subjected to SDS-PAGE. The proteins were transferred onto nitrocellulose membranes (GE Healthcare sciences). The transferred membranes were blocked using TBST with 5% w/v nonfat dry milk and incubated with indicated primary antibodies at 4 °C overnight. The antibodies used: SPOP (dilution 1:1000, 16750-1-AP, Proteintech), GLP (dilution 1:1000, A301-642A, Bethyl), G9a (dilution 1:1000, PP-A8620A-00, R&D), MYC (dilution 1:1000, M192-7, MBL), Flag (dilution 1:1000, F180-4, Sigma), HA (dilution 1:1000, M180-7, MBL), Actin (dilution 1:1000, AC028, Abclonal), DNMT1 (dilution 1:1000, 5032 S, CST), DNMT3A (dilution 1:300, SC-373905, Santa Cruz Biotechnology), DNMT3B (dilution 1:300, SC-376043, Santa Cruz Biotechnology), DEK (dilution 1:1000, 13962 S, CST), BRD4 (dilution 1:1000, 13440 S, CST), CUL3 (dilution 1:1000, 2759 S, CST), RBX1 (dilution 1:1000, PA5-29149, Thermo Scientific), FOXO3A (dilution 1:1000, 12829 S, CST), GATA5 (dilution 1:300, SC-373684, Santa Cruz Biotechnology), Histone H3 (dilution 1:3000, 9715, CST), H3K9 2me (dilution 1:1000, ab1220, Abcam), NDRG1 (dilution 1:300, SC-398291, Santa Cruz Biotechnology), AR (dilution 1:1000, SC-815, Santa Cruz Biotechnology), PD-L1 (dilution 1:1000, 13684S, CST), cleaved-PARP (dilution 1:1000, 5625S, CST). The second day, the membranes were washed 3 times with TBST and followed by incubation with secondary antibodies at room temperature. After washing in TBST for three times, the membranes were visualized using Enhanced Chemiluminescence (ECL) system (Thermo Fisher Scientific) and exposed to X-ray films.

### In vivo ubiquitination assay

293T cells were transfected with HA–ubiquitin and the indicated plasmids. 36 h after transfection, cells were treated with 20 μM MG132 for 8 h and lysed in 1% SDS sample buffer (50 mM Tris pH 7.5, 0.5 mM EDTA, 1 mM DTT) and boiled for 10 min. For immunoprecipitation, the cell lysates were diluted 10-fold in Tris-HCl buffer and incubated with anti-Flag M2 agarose beads (Sigma) for 4 h at 4 °C. The bound beads are washed four times with BC100 buffer (20 mM Tris-Cl, pH 7.9, 100 mM NaCl, 0.2 mM EDTA, 20% glycerol) containing 0.2% Triton X-100. The protein was eluted with 3 × Flag peptide for 2 h at 4 °C. The ubiquitinated form of GLP was detected by western blot using anti-HA antibody.

### RNA extraction and reverse transcription-quantitative PCR (RT-qPCR)

The total RNA was isolated using TRIzol reagent (Thermo Fisher Scientific) and reverse-transcribed to cDNA using superscript RT kit (Promega GoScript) according to manufacturer’s instruction. Quantitative PCR was performed using SYBR Green Master mix Kit (Bio-Rad) in Bio-Rad CFX manager 3.1. The quantification of indicated genes was normalized to that of endogenous control *GAPDH*. The primers for RT-qPCR are listed in Supplementary Data 11.

### Cell proliferation assay

Cells were seeded in 96-well plates in a concentration of 2000 cells per well. After cells adhered to the well bottom, indicated drugs were administrated to the wells to treat the cells. At indicated time points, the CellTiter 96 Aqueous One solution Cell Proliferation Assay (MTS) (Promega) was used to measure cell viability. MTS was diluted at a ratio of 1:10 in PBS and added into the wells and incubated for 1 h at 37 °C in a cell incubator. Microplate reader was used to measure absorbance of 490 nm in each well.

### Colony formation assay

The procedure was carried out by seeding 4 × 10^3^ cells onto each well of 6-well plate. Approximately 12 days later, the colonies were fixed with 4% paraformaldehyde for 15 min and stained with crystal violet (0.5% w/v) for 1 h. The colonies were gently washed with running tap water. The colonies with more than 50 cells were counted.

### Migration assay and wound-healing assay

Cell migration was determined by Transwell (Costar) migration assays. Briefly, 3 × 10^4^ 22Rv1 cells were seeded in serum-free medium in the upper chamber, and the lower chamber was filled with RPMI 1640 containing 10% FBS. After cultured for 48 h, migrated cells were fixed and stained with 0.1% crystal violet and counted and imaged under microscope. For wound-healing assay, 22Rv1 cells were seeded in 6-well plates. When cells were grown to 90% confluence, the wounds were created by tips. The floating cells were removed by changing fresh media. Images were taken after 24 h, and the wound closure ratios were calculated by measuring the area recovered as a percentage of the original area.

### Immunohistochemistry (IHC)

Formalin-fixed paraffin-embedded (FFPE) tumor tissues were sectioned at 4 µm thickness and heated at 65 °C for 1 h before deparaffinized two times in xylene and rehydrated two times with absolute ethanol, 95% ethanol, 70% ethanol, and water. The sections were immersed in 0.45% H_2_O_2_ in methanol for 30 min to quench endogenous peroxidase activity followed by heat-induced antigen retrieval using unmasking solution (Vector Labs) in microwave oven for 20 min. Cooling to room temperature, for 5mC IHC, the tissue sections were treated with 2 N HCl for 30 min at room temperature. The slides were blocked with normal goat serum PBS/FSGO solution (4 drops of Avidin blocking solution (Vector Labs) in 1 ml PBS containing 0.5% FSGO) and incubated with primary antibody at 4 °C overnight. IHC of tumor samples was performed using primary antibodies against G9a (dilution 1:1000; R&D, PP-A8620A-00), GLP (dilution 1: 600; Abcam, ab241306), Ki-67 (dilution 1:10000, Abcam, ab15580) and 5mC (dilution 1:1000; Abcam, ab10805). The slides were washed three times in PBS/FSGO and incubated with biotinylated secondary antibody for 1 h at room temperature. After incubation, the slides were washed three times in PBS/FSGO and incubated with ABC solution for 30 min, followed by DAB development (DAB-2031, Millipore sigma). Hematoxylin and Scott’s water were used for bluing nucleus followed by dehydration with ethanol and xylene. Images were acquired using Leica microscope. Informed consent was obtained from all human participants, and the studies were approved by the Institute Review Board (IRB) of the Mayo Clinic.

### Immunofluorescence cytochemistry using cultured cells

22Rv1 cells were seeded on coverslips in a 6-well plate. Transfection was performed after cells were adherent to the coverslips. 24 h after transfection, cells on coverslips were washed with PBS and fixed with 4% paraformaldehyde in PBS for 20 min. Specimens were washed with PBS for three times. For 5mC staining, the specimens were treated with 2 N HCl for 30 min. Specimens were permeabilized with 0.2% triton in PBS for 15 min and blocked with blocking buffer (5% glycerol, 5% goat serum in PBS) for 1 h in room temperature, followed by incubation with primary antibody overnight at 4°C. After washed three times with PBS, specimens were incubated with fluorochrome-conjugated secondary antibody (Invitrogen Alexa Fluor 488/594) for 1 h with protection from light. Coverslips were washed three times with PBS and mounted with VECTASHIELD mounting medium. Images were acquired using Zeiss LSM 780 confocal microscope.

### Prostate cancer xenograft and drug treatment

The animal study was approved by the Institutional Animal Care and Use Committee (IACUC) at the Mayo Clinic. Six-week-old SCID male mice were housed in standard condition with a 12-h light /12-h dark cycle and randomly divided into different groups as indicated. 5 × 10^6^ of DU145 cells infected with lentivirus expressing EV or HA-SPOP-F102C were mixed with Matrigel (50 μl of PBS plus 50 μl of Matrigel (BD Biosciences)) and injected subcutaneously into mice. When xenografts reached a size of approximately 100 mm^3^, indicated vehicle (Saline, 5% DMSO, 30% PEG 300 and 5% Tween 80) and drugs (Docetaxel, 5 mg/kg (in 5% DMSO, 30% PEG 300 and 5% Tween 80); 5-AzaC, 2 mg/kg (in saline)) were administered individually or in combination by intraperitoneal (i.p.) injection every other day. Tumor growth was measured in a blinded fashion by calipers. The volume of the tumors was calculated using the formula (*L* × *W*^*2*^)/2, where *L* stands for the length of the tumor and W stands for the width. Tumor volumes were compared, and *P*-values were determined by Student’s *t* test. 28 days after cell injection, the tumors were dissected and photographed.

### Genome-wide DNA methylation profiling using MethylationEPIC BeadChip

Genome-wide DNA methylation profiling was performed using the Illumina Infinium MethylationEPIC Beadchip array by the University of Minnesota Genomic Center (UMGC). Genomic DNA of 22Rv1 cells expressing empty vector (EV) or HA-SPOP-F102C was extracted using a standard DNA extraction method. Briefly, cells were digested with 20 μg/ml proteinase K in reaction buffer (50 mM Tris pH 8.0, 50 mM EDTA, 100 mM NaCl, 0.5% SDS) overnight and extracted with phenol/chloroform isoamyl alcohol followed by ethanol precipitation and resolved in low TE buffer (10 mM Tris pH 8.0, 0.1 mM EDTA). Based on PicoGreen quantification, a total of 250 ng of each sample was bisulfite converted using the EZ DNA Methylation Kit. Bisulfite-treated samples were then amplified, fragmented, purified and hybridized onto the EPIC Beadchip according to the manufacturer’s standard protocol. The arrays were washed and scanned using the Illumina HiScan System. Quality control report of the hybridization was shown in Supplementary Data 2–4.

### Analysis of methylationEPIC Beadchip data

Differentially methylated CpGs (adjusted *p*-value < 0.05) between the F102C-expressing cells and the EV control cells were identified using the T-test, and the Benjamini–Hochberg procedure was used to adjust the raw p-values for multiple test correction. Heatmap of differentially methylated CpGs was generated by Mev (http://mev.tm4.org/) after converting methylation beta values into Z-scores. The stacked barplot, bean plot, and the 2D PCA plots were generated by CpGtools^54^. Specifically, all the CpGs were used for the stacked barplot and the bean plots, but only the top 10,000, 20,000, and 50,000 most variable CpGs were selected for PCA analyses.

### Analysis of whole-genome bisulfite sequencing (WGBS) data in castration-resistant prostate cancer patient samples

Whole-genome bisulfite sequencing (WGBS) data from West Coast Prostate Cancer Dream Team (WCDT) were originally generated in the lab of Dr. Felix Feng^7^. Homo sapiens (human) genes (GRCh38) were annotated using R package biomaRT^55^. WGBS data were visualized using R package Gviz^56^. 100 WCDT samples were divided into two groups based on SPOP mutation status (“WT” versus “MUT”) where 5 out of 100 WCDT cases harbor SPOP mutation. The average β value of each group was used as the overall methylation level. Slide window smoothing was performed with a window size of 300 bp using aggregation method (parameters: window = −1, window Size = 300).

### Methylation-specific PCR

Genomic DNA extraction was performed as described above. A total of 2 μg genomic DNA of each sample was used for incubation in 18 μl of reaction buffer (2 μg tRNA, 280 μg/ul proteinase K, 1% SDS) for 1 h in 37 °C and denatured in 0.3 M NaOH in 95 °C for 2 min. The denatured DNA was subjected to saturated metabisulphite with 0.5 mM Quinol and incubated at 55 °C overnight for bisulfite deamination. The samples were desalted through desalting column and desulphonated in 0.3 M NaOH at 37 °C for 5 min and neutralized in 3 M NH_4_OAc followed by ethanol precipitation to purify DNA. The recovered DNA was used as template for PCR amplification using conversion-specific primers. The methylation-specific primers are listed in Supplementary Data 11.

### DNA dot blot

Genomic DNA was denatured in 95 °C for 5 min and spotted on nylon membranes followed by crosslink. One copy of spotted membrane was subjected to methylene blue staining. The other was blocked with 5% skimmed milk and incubated with anti-5mC antibody overnight at 4 °C and HRP-conjugated secondary antibody for 1 h at RT. After washing in TBST for three times, the membranes were visualized using Enhanced Chemiluminescence (ECL) system (Thermo Fisher Scientific) and exposed to X-ray films.

### Methylated DNA immunoprecipitation (MeDIP) and quantitative PCR

Genomic DNA extraction was performed as described above. A total of 1 μg genomic DNA of each sample was subjected to sonication for 7 min on automatic setting (30 s on 30 s off at maximum power). The sonicated DNA was denatured in 95 °C for 10 min and cool immediately on ice. Antibody specific to methylated cytosine and protein A/G beads were added together with IP buffer to the denatured DNA to a final volume of 500 μl and incubated for 2 h at 4 °C in rotating holder. The beads were washed with IP buffer three times and re-suspended in 400 μl of digestion buffer followed by proteinase K digestion overnight at 50 °C. The DNA bound with beads was isolated by Phenol/chloroform and centrifuged. The supernatant was transferred to another tube followed by ethanol precipitation (adding 1/10 volume of 3 M sodium acetate, 1 μl of glycogen (20 μg/μl), and 2 volumes of absolute ethanol and placed at −20 °C for 20 min). The samples were centrifuged and washed with 70% ethanol, and re-suspended in 10 μl H_2_O for qPCR analysis. The primers for MeDIP qPCR are listed in Supplementary Data 11.

### Proximity ligation assay (PLA)

22Rv1 cells were seeded into 24 well chamber slides. After 24 h in DMEM, the cells were transfected with Flag-GLP and Myc-SPOP plasmids. 24 h after transfection, cells were fixed with 4% paraformaldehyde. Cells were then permeabilized in 0.4% Triton X-100 and blocked in Duolink Blocking buffer (Sigma) for 1 h at 37°C. For the in situ PLA, we used the Duolink in situ Red kit (Sigma-Aldrich, DUO92101). Primary antibodies with anti-Flag and anti-Myc were incubated overnight at 4 °C. The next day, Plus and Mines PLA probes were incubated for 1 h at 37 °C. Ligation and amplification of the PLA were performed using the Duolink In Situ Detection Reagents Red (Sigma). After several washes, cells were mounted in Prolong Gold mounting media with DAPI. Cells were imaged using a confocal microscope (LSM880, Zeiss) with a 63*/1.4NA Oil PSF Objective.

### Statistical analysis

All data are shown as means ± SD. for experiments performed with three replicates unless otherwise specified. The data were processed in Microsoft Excel version 2008. Differences between the two groups were analyzed using unpaired Student’s *t* tests unless otherwise specified. A *P*-value < 0.05 was considered statistically significant.

## Supplementary information

Supplementary Information

Description of Additional Supplementary Files

Dataset 1

Dataset 2

Dataset 3

Dataset 4

Dataset 5

Dataset 6

Dataset 7

Dataset 8

Dataset 9

Dataset 10

Dataset 11

Dataset 12

## Data Availability

The MethylationEPIC BeadChip data have been deposited to the National Center for Biotechnology Information (NCBI) Gene Expression Omnibus (GEO) database with the accession code GSE179234. All relevant data are available from the authors. Source data are provided with this paper.

## Source data

Source Data
