# Supplementary material for: SPOP mutation induces DNA methylation via stabilizing GLP/G9a
Source: Nat Commun. 2021 Sep 29;12:5716. doi: 10.1038/s41467-021-25951-3 (PMC8481544; doi:10.1038/s41467-021-25951-3)
Supplement: Supplementary file 1 — Supplementary Information [file 41467_2021_25951_MOESM1_ESM.pdf]

**Supplementary Information**

# SPOP mutation induces DNA methylation via stabilizing GLP/G9a

Jianong Zhang, Kun Gao, Hongyan Xie, Dejie Wang, Pingzhao Zhang, Ting Wei, Yuqian Yan, Yunqian Pan, Wenbin Ye, Huifen Chen, Qing Shi, Yao Li, Shi-min Zhao, Xiaonan Hou, Saravut J. Weroha, Yuzhuo Wang, Jun Zhang, R. Jeffrey Karnes, Housheng Hansen He, Liguo Wang, Chenji Wang and Haojie Huang

Supplementary of figure 1

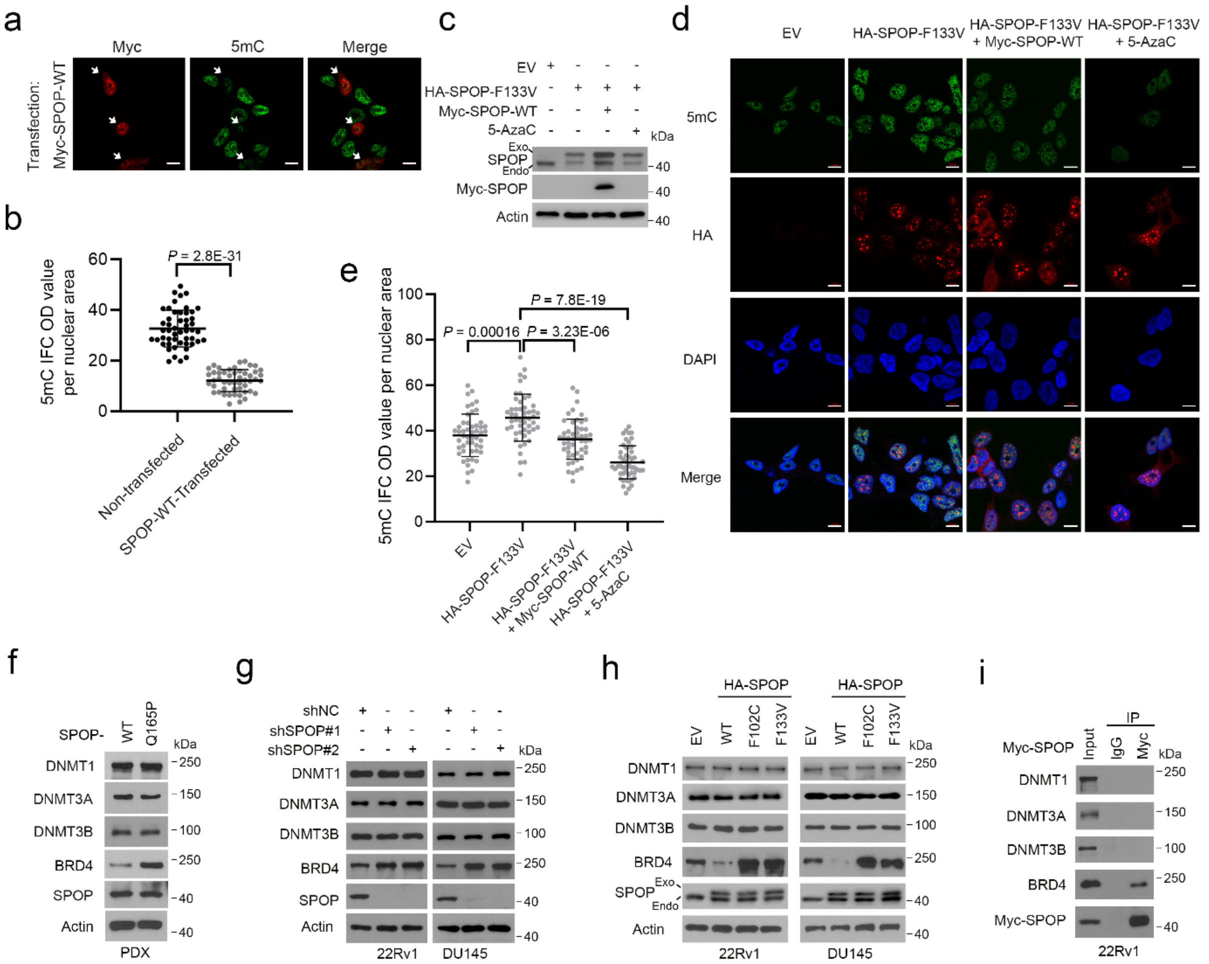


**Supplementary Figure 1. SPOP regulates DNA methylation without affecting DNMT protein level.**

(**a, b**) Representative IFC images of Myc-SPOP and 5mC staining in 22Rv1 cell transfected with Myc-SPOP WT (**a**). Scale bar, 10 μm. Arrows indicate the Myc-SPOP WT-transfected cells. ImageJ was used to quantify the optical density (OD)/nuclear area (pixel) of 5mC staining in SPOP-transfected and non-transfected cells (**b**). Data shown means ± SD (n = 50 cells/group). (**c-e**) 22Rv1 cells stably expressing EV, SPOP-F133V were transfected with EV, SPOP-WT or treated with 2 μM 5-AzaC for 48 h. Cells were used for western blots with indicated antibodies (**c**) and IFC staining of HA-SPOP F133V and 5mC (**d**). Scale bar, 10 μm. ImageJ was used to

quantify the optical density (OD)/nuclear area (pixel) of 5mC staining in 4 groups(**e**). Data shown means ± SD (n = 50 cells/group).

(**f**) Western blots in whole cell lysate (WCL) from SPOP wild type (WT) and Q165P mutant PDX tumors. BRD4 was used as a positive control.

(**g**) Western blots in WCL from 22Rv1 and DU145 cells infected with lentivirus expressing negative control (shNC) or SPOP-specific shRNAs.

(**h**) Western blots in WCL from 22Rv1 and DU145 cells infected with lentivirus expressing empty vector (EV), WT SPOP or mutants F102C or F133V.

(**i**) Western blots of WCL and co-IP samples of anti-Myc antibody from 22Rv1 cells transfected with Myc-tagged SPOP. Statistical significance was determined by unpaired two-tailed Student's *t*-test in **b**, **e**. Experiments in **f**, **g**, **h**, **i** were repeated twice. Source data are provided as a Source Data file.

Supplementary figure 2

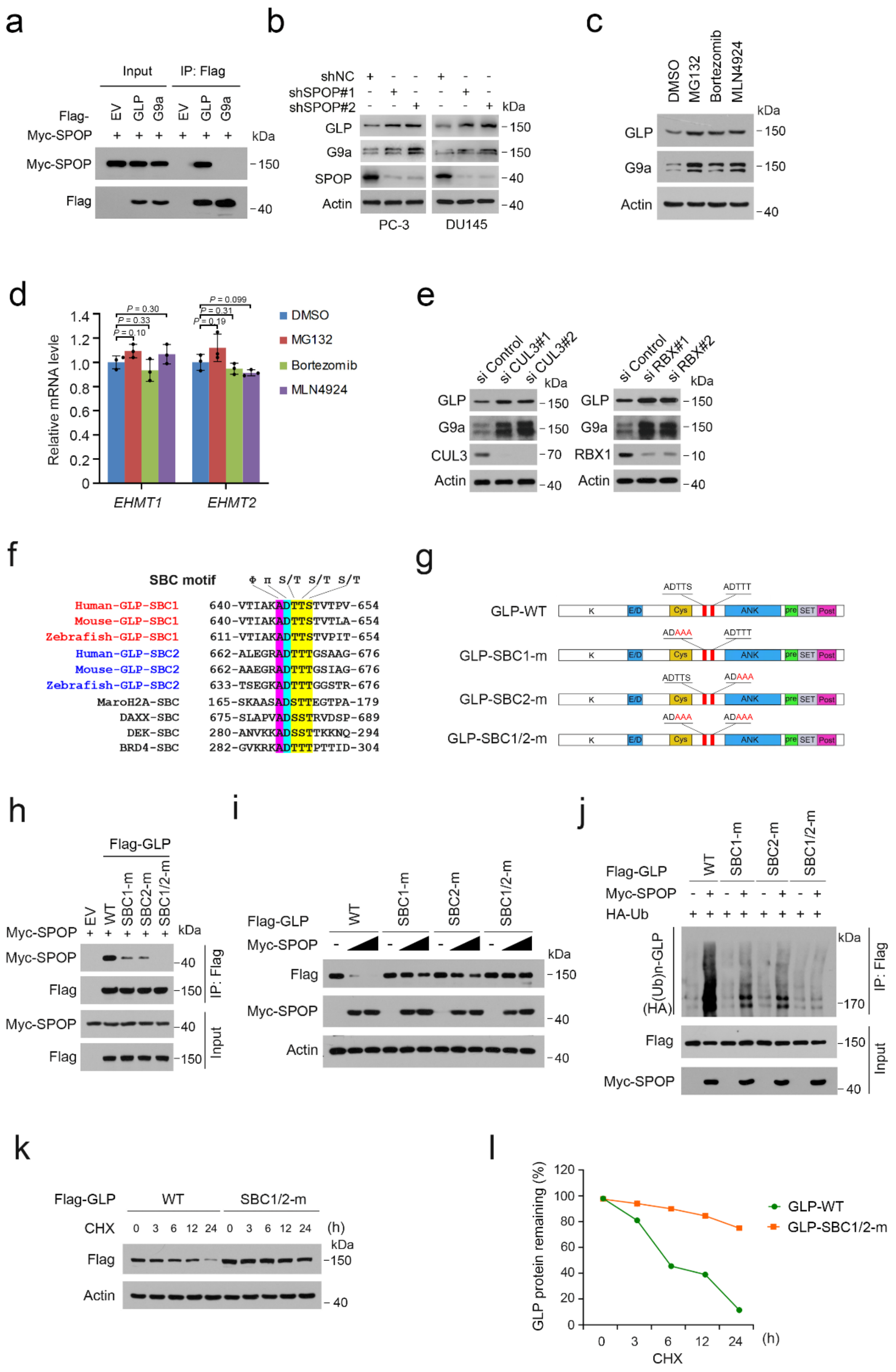

**Supplementary Figure 2. SPOP promotes GLP protein degradation via two SBC motifs.**

(**a**) Western blots of WCL and co-IP samples of anti-Flag antibody from 293T cells transfected with indicated plasmids and treated with 20 μM MG132 for 8 h.

(**b**) Western blots of WCL from PC-3 and DU145 cells infected with lentivirus expressing negative control shRNA (shNC) or SPOP-specific shRNAs.

(**c, d**) Western blots of WCL (**c**) and RT-qPCR measurement of mRNA level of *EHMT1* and *EHMT2* genes (**d**) from 22Rv1 cells treated with DMSO, MG132 (20 μM), bortezomib (200 nM) or MLN4924 (200 nM) for 8 h. Data shown means ± SD (n = 3 replicates/group).

(**e**) Western blots of WCL from 22Rv1 cells transfected with indicated siRNAs for 48 h.

(**f**) Amino acid sequence alignment of two potential SBC motifs of GLP in human, mouse and zebrafish and a few known SPOP substrates.

(**g**) Diagram showing wild-type and SBC motif-mutated GLP constructs.

(**h**) Western blots of WCL and co-IP samples of anti-Flag antibody from 293T cells transfected with the indicated plasmids and treated with 20 μM MG132 for 8 h.

(**i**) Western blots of WCL from 293T cells transfected with the indicated plasmids.

(**j**) Western blots of WCL and IP samples of anti-Flag antibody from 293T cells transfected with the indicated plasmids and treated with 20 μM MG132 for 8 h.

(**k, l**) Western blots of WCL of 293T cell transfected with indicated plasmids and treated with 50 μg/ml of CHX and harvested at different time points (**k**). Western blots were quantified and at each time point, the intensity of GLP protein was normalized to the intensity of actin and then to the value at 0-h time point (**l**). Statistical significance was determined by unpaired two-tailed Student's *t*-test in **d**. Experiments in **a**, **b**, **e**, **h**, **i**, **j**, were repeated twice. Source data are provided as a Source Data file.

Supplementary figure 3

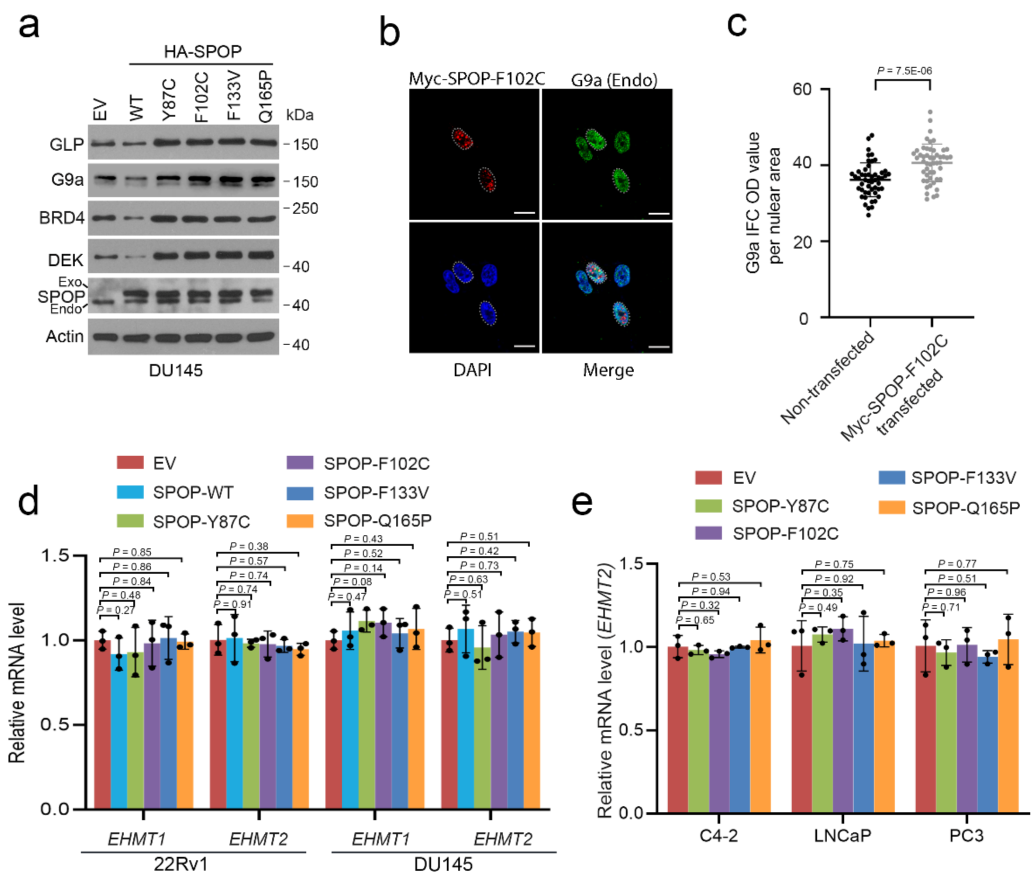


**Supplementary Figure 3. GLP and G9a expression in SPOP mutant PCa cell lines.**

(**a**) Western blots of WCL from DU145 cells infected with lentivirus expressing empty vector (EV), SPOP WT or mutants.

(**b, c**) Representative IFC images of Myc-SPOP-F102C and endogenous G9a staining in 22Rv1 cells transfected with Myc-SPOP-F102C (**b**). Scale bar, 10 µm. ImageJ was used to quantify the optical density (OD)/nuclear area (pixel) of G9a staining in SPOP-transfected and non-transfected cells (**c**). Data shown means ± SD (n = 50 cells/group).

(**d**) RT-qPCR measurement of mRNA expression of *EHMT1* and *EHMT2* genes in 22Rv1 and DU145 cells infected with lentivirus expressing EV, SPOP WT or mutants. Data shown means ± SD (n = 3 replicates/group).

(**e**) RT-qPCR measurement of mRNA expression of *EHMT2* in C4-2, LNCaP and PC3 cells infected with lentivirus expressing EV or SPOP mutants. Data shown means ± SD (n = 3 replicates/group). Statistical significance was determined by unpaired two-tailed Student's *t*-test in **c**, **d**, **e**. Experiments in **a** were repeated twice. Source data are provided as a Source Data file.

# Supplementary figure 4

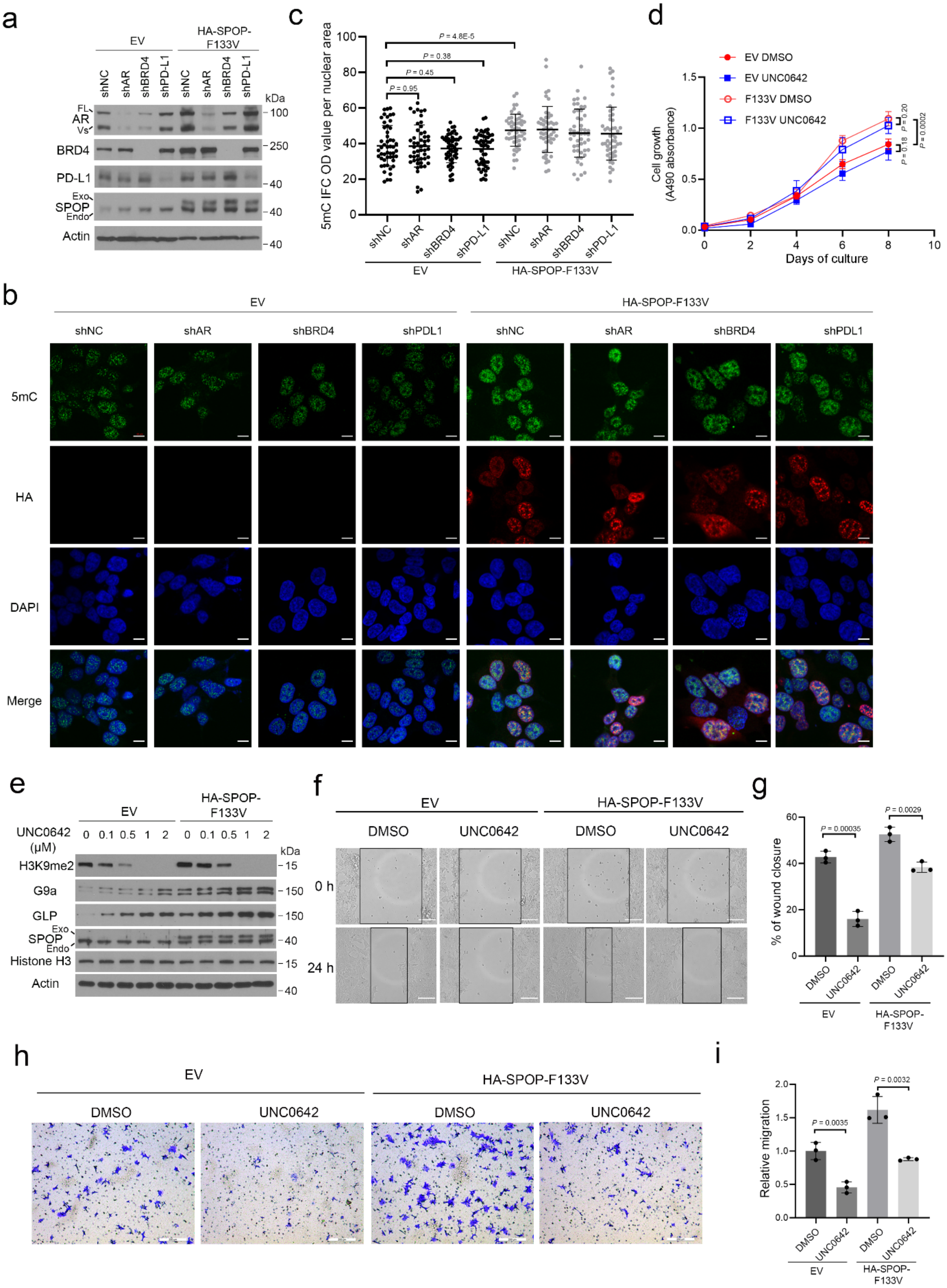

**Supplementary Figure 4. SPOP mutation-induced DNA hypermethylation is independent of AR, BRD4 and PD-L1 and increased H3K9me2 affects cell migration but not proliferation.**

(**a-c**) 22Rv1 cells were infected with lentivirus expressing empty vector (EV) and SPOP-F133V and indicated shRNAs. Cells were subjected to western blots (**a**) and IFC staining 5mC and HA-SPOP-F133V (**b**). Scale bar, 10 μm. ImageJ was used to quantify the optical density (OD)/nuclear area (pixel) of 5mC staining (**c**). Data shown means ± SD (n = 50 cells/group).

(**d**) MTS assay in 22Rv1 cells stably expressing EV or SPOP-F133V and/or treated with DMSO or 2 μM UNC0642. Data shown means ± SD (n = 5 replicates/group).

(**e**) Western blots of WCL from 22Rv1 cells infected with lentivirus expressing EV or SPOP-F133V and/or treated with different doses of UNC0642 for 24 h.

(**f, g**) Representative images of wound healing assay in 22Rv1 cells infected with lentivirus expressing EV or SPOP-F133V and/or treated with 2 μM UNC0642 for 24 h (**f**). Scale bar, 100 μm. Quantification of wound closure by measuring the area recovered as a percentage of the original area (**g**). Data shown means ± SD (n = 3 replicates/group).

(**h, i**) Representative images of transwell migration assay using 22Rv1 cells infected with lentivirus expressing EV or SPOP-F133V and/or treated with 2 μM UNC0642 for 24 h (**h**). Scale bar, 200 μm. Quantification of transwell migration assay by counting the migrated cell number and then cell number in each group was normalized to that of the control group (**i**). Data shown means ± SD (n = 3 replicates/group). Statistical significance was determined by unpaired two-tailed Student's *t*-test in **c**, **d**, **g**, **i**. Experiments in **e** were repeated twice. Source data are provided as a Source Data file.

Supplementary figure 5

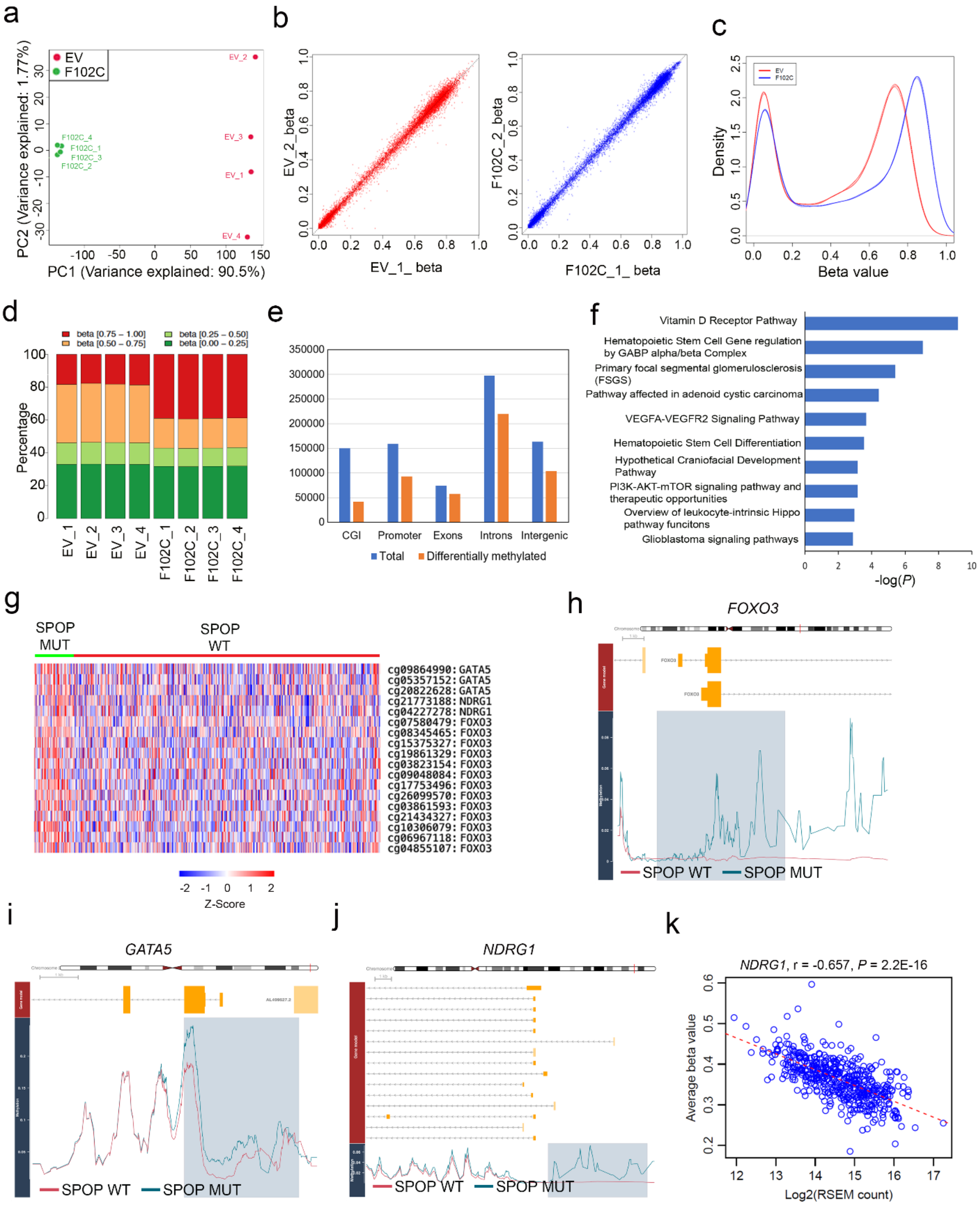

**Supplementary Figure 5. Identification of hypermethylated genes in SPOP mutated PCa cells by MethylationEPIC BeadChip.**

(**a**) PCA plot using the top 20,000 most variable CpGs ranked by the standard deviation in replicates of 22Rv1 cells stably expressing EV or SPOP-F102C.

(**b**) Representative scatter plot showing the reproducibility of two biological replicates from the EV (left) and F102C (right) groups. Detailed information regarding the Pearson correlation of all four replicates in both EV and F102C groups is shown in Supplementary Data 4.

(**c**) Density plot of beta values for EV (red) and F102C (blue) groups, each line represents a biological replicate.

(**d**) Stacked bar plot showing the distribution of beta values in four bins in each replicate.

(**e**) Genomic distribution of all CpGs (blue) and the differentially methylated CpGs (orange) identified by MethylationEPIC BeadChip.

(**f**) Pathway analysis of 115 TSGs which were hypermethylated and downregulated in SPOP mutant PCa patient specimens compared to SPOP WT samples in the TCGA database.

(**g**) Heat map shows differential DNA methylation levels at *FOXO3*, *NDRG1* and *GATA5* gene loci in SPOP WT and mutant patient samples revealed by the TCGA PCa 450K methylation data.

(**h-j**) A plot with sliding window value to show the methylation level in the promoter region (highlighted) of *FOXO3* (**h**), *GATA5* (**i**), and *NDRG1* (**j**) genes in SPOP mutant (SPOP MUT) patient samples compared to SPOP WT counterparts in a cohort of mCRPC patients (see reference 7 in the main text).

(**k**) The correlation between promoter methylation and expression of *NDRG1* gene in the TCGA PCa database. Statistical significance was determined by unpaired two-tailed Student's *t*-test.

# Supplementary figure 6

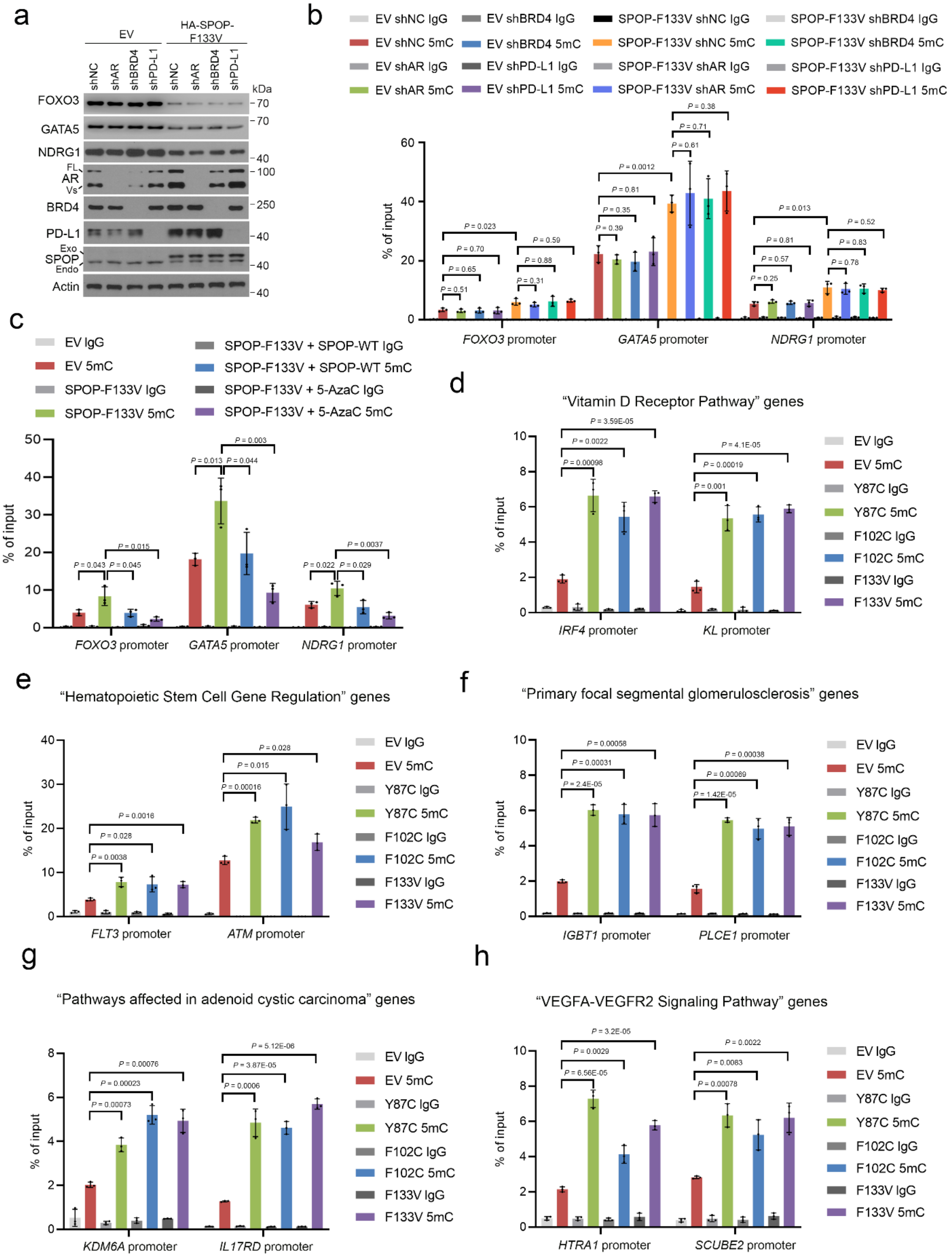

**Supplementary Figure 6. SPOP mutation-caused promoter hypermethylation is independent of AR, BRD4 and PD-L1 but can be reversed by SPOP WT or 5-AzaC and validation of promoter hypermethylation of genes from different pathways induced by SPOP mutations.**

(**a, b**) 22Rv1 cells infected with lentivirus expressing indicated plasmids and shRNAs were harvested for western blots analysis with indicated antibodies (**a**) or for MeDIP-qPCR to detect the promoter methylation of the indicated target genes (**b**). Data shown means ± SD (n = 3 replicates/group).

(**c**) 22Rv1 cells expressing EV or SPOP-F133V were transfected with SPOP-WT or treated with 2 μM 5-AzaC for 48 h and cells were harvested for MeDIP-qPCR to detect the methylation changes in promoter of the indicated target genes. Data shown means ± SD (n = 3 replicates/group).

(**d-h**) 22Rv1 cells expressing empty vector (EV) or different SPOP mutants were used for MeDIP-qPCR to validate the methylation changes in the promoter of the indicated target genes in the pathways of "vitamin D receptor pathway" (**d**), "hematopoietic stem cell gene regulation" (**e**), "primary focal segmental glomerulosclerosis" (**f**), "pathways affected in adenoid cystic carcinoma" (**g**), and "VEGFA-VEGFR2 signaling pathway" (**h**). Data shown means ± SD (n = 3 replicates/group). Statistical significance was determined by unpaired two-tailed Student's *t*-test in **b**-**h**. Source data are provided as a Source Data file.

## Supplementary figure 7

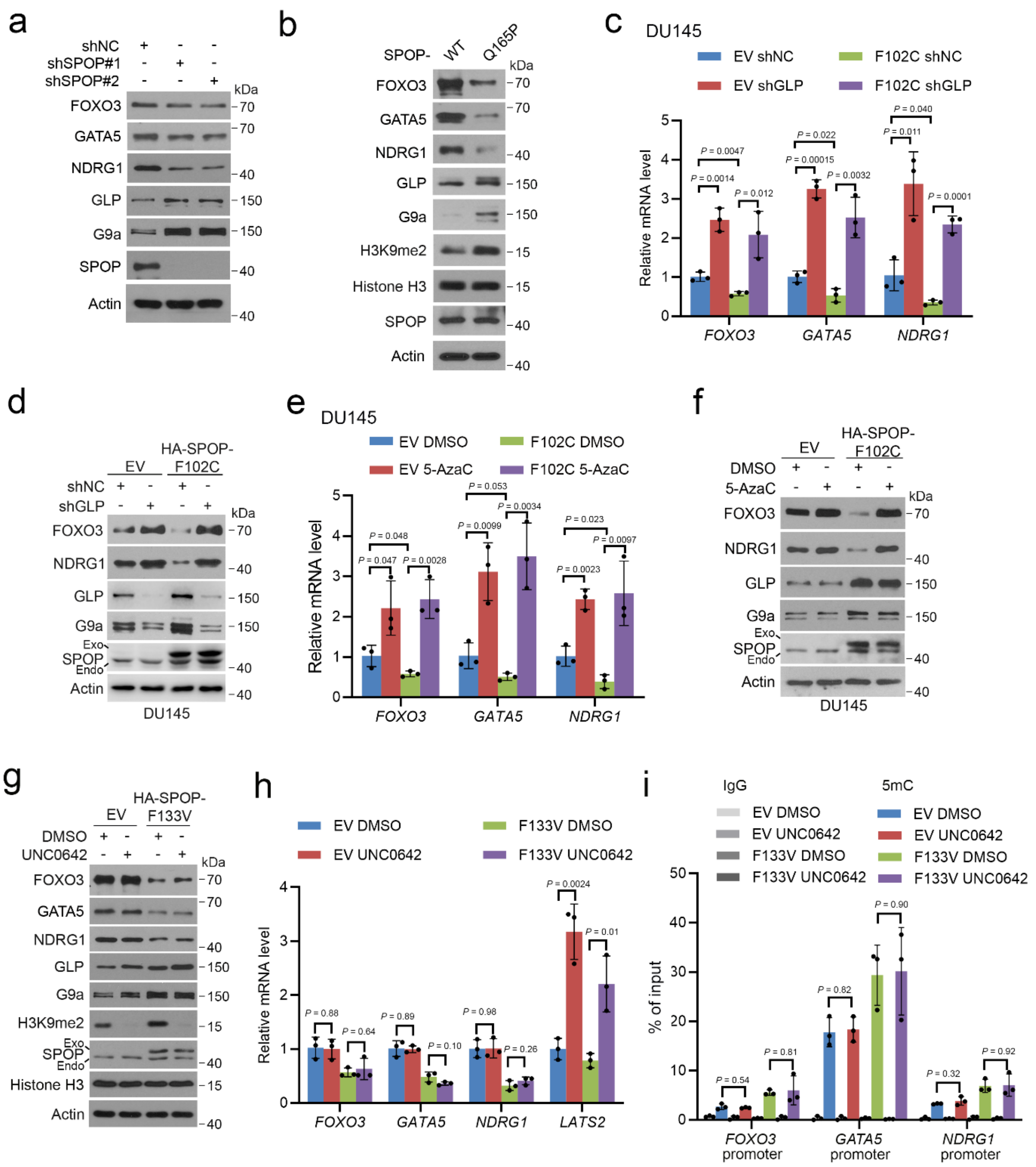


**Supplementary Figure 7. Effect of SPOP knockdown and mutation on expression of genes regulated by the GLP independent of its enzymatic activity.**

(**a**) Western blots of WCL from 22Rv1 cells infected with lentivirus expressing negative control shRNA (shNC) or SPOP-specific shRNAs.

(**b**) Western blots of WCL from SPOP WT or Q165P mutant PDX tumor tissues.

(**c, d**) RT-qPCR measurement of mRNA level of indicated genes (**c**) and Western blots of WCL (**d**) in DU145 cells infected with lentivirus expressing indicated plasmids and/or shRNAs. EV, empty vector. Data shown means ± SD (n = 3 replicates/group).

(**e, f**) RT-qPCR measurement of mRNA level of indicated genes (**e**) and Western blots of WCL (**f**) in DU145 cells infected with lentivirus expressing indicated plasmids and/or treated with DMSO or 2 μM 5-AzaC for 48 h. Data shown means ± SD (n = 3 replicates/group).

(**g-i**) 22Rv1 cells expressing EV or SPOP F133V were treated with DMSO or 2 μM UNC0642 for 24 h. Cells were harvested for western blots (**g**), RT-qPCR (**h**) and MeDIP-qPCR (**i**). Data shown means ± SD (n = 3 replicates/group). Statistical significance was determined by unpaired two-tailed Student's *t*-test in **c**, **e**, **h**, **i**. Experiments in **a**, **b** were repeated twice. Source data are provided as a Source Data file.

## Supplementary figure 8

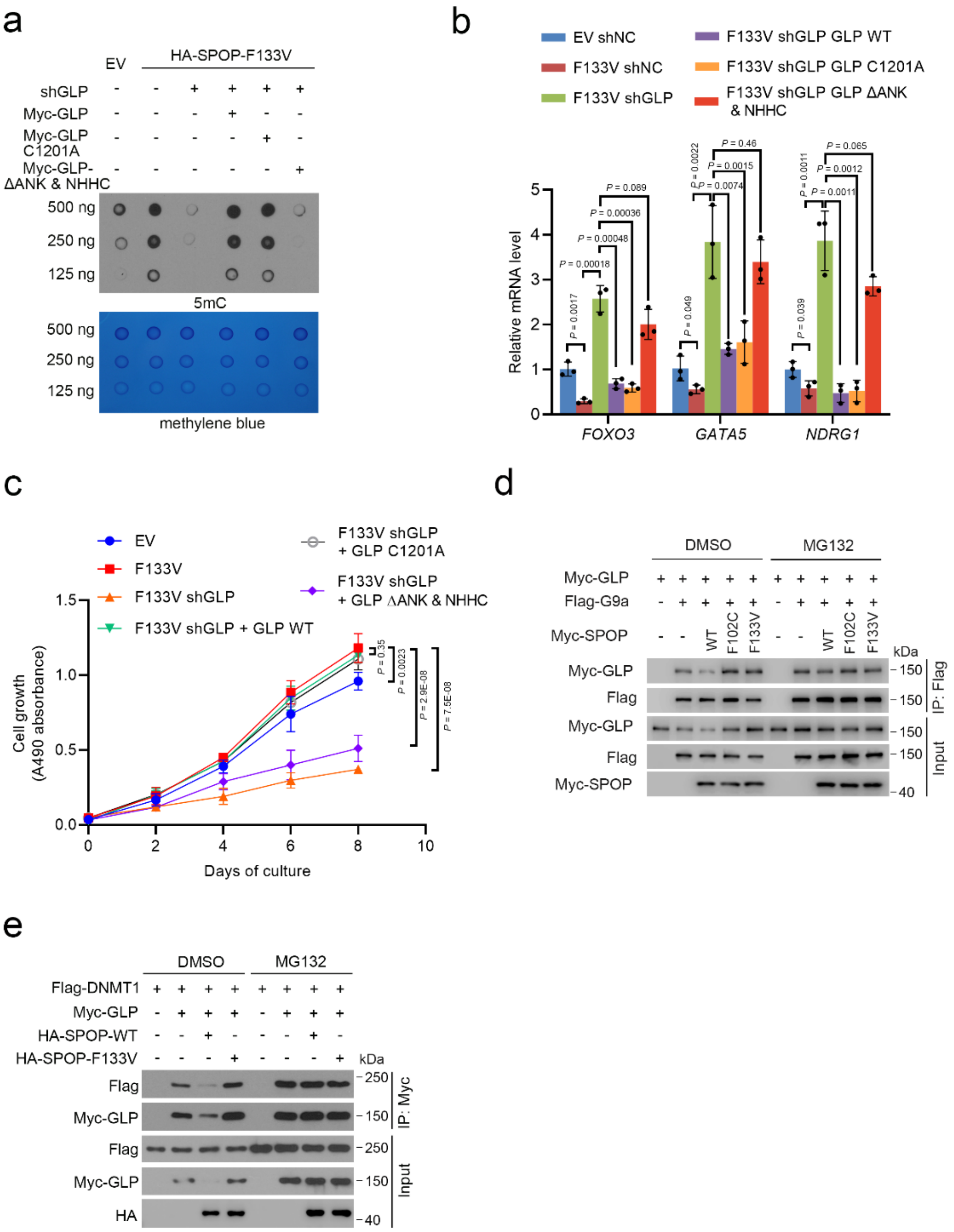


**Supplementary Figure 8. DNMT interaction but not the enzymatic activity of GLP is essential for SPOP mutation-induced DNA methylation and gene silence, and SPOP mutation does not affect the binding affinity between GLP and G9a or GLP and DNMT1.**

(**a**) 22Rv1 cells expressing indicated shRNA and/or plasmids were harvested for DNA isolation and dot blot analysis using 5mC antibody. Methylene blue staining was performed to control the DNA loading.

(**b**) 22Rv1 cells infected with lentivirus expressing indicated plasmids and/or shRNAs and transfected with indicated constructs were used for RT-qPCR. Data shown means ± SD (n = 3 replicates/group).

(**c**) MTS assay in 22Rv1 cells expressing indicated shRNA and/or plasmids. Data shown means ± SD (n = 5 replicates/group).

(**d**) Western blots of WCL and co-IP samples of anti-Flag antibody from 293T cells transfected with indicated plasmids and/or treated with DMSO or 20 μM MG132 for 8 h.

(**e**) Western blots of WCL and co-IP samples of anti-Myc antibody from 293T cells transfected with indicated plasmids and/or treated with DMSO or 20 μM MG132 for 8 h. Statistical significance was determined by unpaired two-tailed Student's *t*-test in **b**, **c**. Experiments in **d**, **e** were repeated twice. Source data are provided as a Source Data file.

Supplementary figure 9

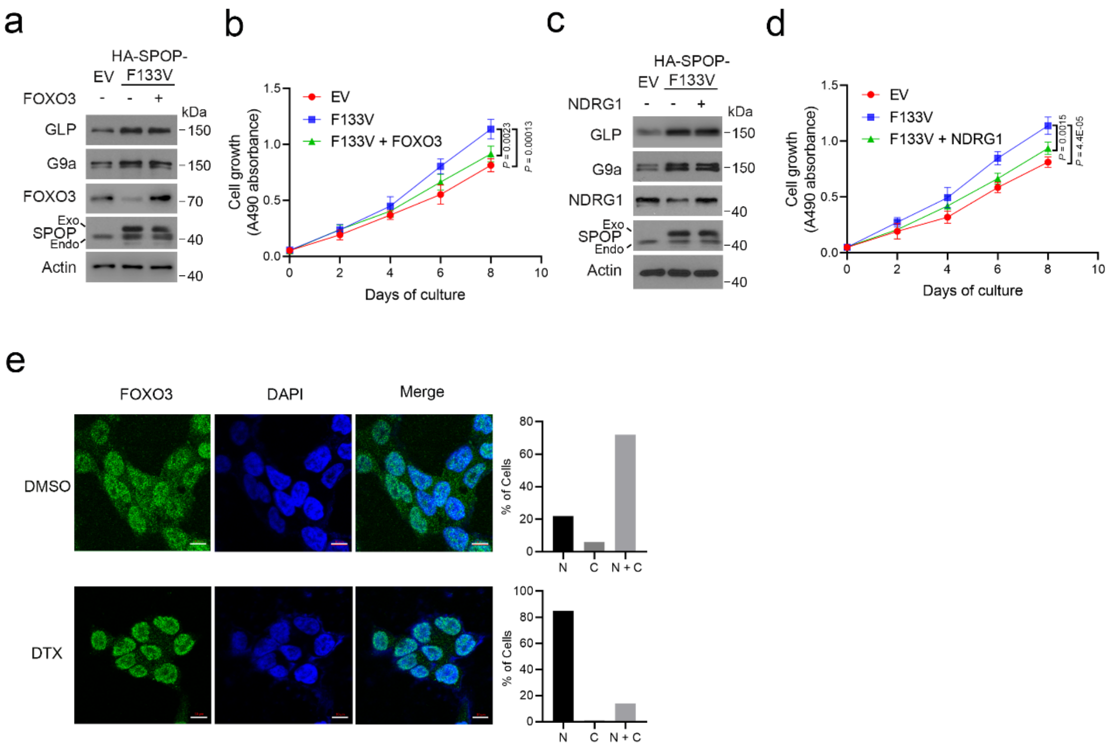


**Supplementary Figure 9. Restored expression of TSG genes repressed by SPOP mutation inhibits growth of SPOP mutant cells and the effect of DTX on FOXO3 cellular localization.** (**a, b**) DU145 cells stably expressing empty vector (EV) or SPOP F133V were transiently transfected with plasmids of EV or FOXO3. Cells were harvested for western blots (**a**) or subjected for MTS assay (**b**). Data shown means ± SD (n = 5 replicates/group).

(**c, d**) DU145 cells stably expressing empty vector (EV) or SPOP F133V were transiently transfected with plasmids of EV or NDRG1. Cells were harvested for western blots (**c**) or subjected for MTS assay (**d**). Data shown means ± SD (n = 5 replicates/group).

(**e**) Representative IFC images of FOXO3 staining in 22Rv1 cells treated with DMSO or DTX for 48 h. Nuclear (N), cytosolic (C) or both nuclear and cytosolic localization (N+C) of FOXO3 protein was quantified. Scale bar, 10 μm. Similar results were obtained from two independent repeats. Statistical significance was determined by unpaired two-tailed Student's *t*-test in **b**, **d**. Source data are provided as a Source Data file.
