# Supplementary material for: SPOP mutation induces DNA methylation via stabilizing GLP/G9a
Source: Nat Commun. 2021 Sep 29;12:5716. doi: 10.1038/s41467-021-25951-3 (PMC8481544; doi:10.1038/s41467-021-25951-3)
Supplement: Supplementary file 2 — Description of Additional Supplementary Files [file 41467_2021_25951_MOESM2_ESM.pdf]

Description of Additional Supplementary Files

File name: Supplementary Data 1

Description: IHC scores of 5mC, GLP and G9a in prostate cancer patient samples with wild-type or mutated SPOP

File name: Supplementary Data 2

Description: Quality control report of Infinium MethylationEPIC BeadChip in empty vector (EV) and SPOP F102C expressing 22Rv1 cells with four replicates per group

File name: Supplementary Data 3

Description: CpG detection rate using Infinium MethylationEPIC BeadChip in control and F102C expressing 22Rv1 cells

File name: Supplementary Data 4

Description: Pearson correlation of replicates in Infinium MethylationEPIC BeadChip

File name: Supplementary Data 5

Description: Hyper methylated CpGs in SPOP F102C expressing  compared to empty vector expressing 22Rv1 cells

File name: Supplementary Data 6

Description: Hyper methylated CpGs in SPOP MUT compared to SPOP WT PCa samples of the TCGA patients

File name: Supplementary Data 7

Description: Shared hyper methylated CpGs between TCGA 450K data in SPOP mutated PCa patient samples and 850K array data in SPOP F102C expressing 22Rv1 PCa cells

File name: Supplementary Data 8

Description: Genes mapped to the shared hyper methylated CpGs between TCGA 450K data in SPOP mutated PCa patient samples and 850K array data in SPOP F102C expressing

File name: Supplementary Data 9

Description: Hypermethylated and downregulated TSGs in SPOP MUT 22Rv1 cells and TCGA PCa patient samples

File name: Supplementary Data 10

Description: Information of antibodies and chemicals

File name: Supplementary Data 11

Description: Information of primer sequences

File name: Supplementary Data 12

Description: Information of shRNA/siRNA sequences
